# RNA Editing Alterations Define Disease Manifestations in the Progression of Experimental Autoimmune Encephalomyelitis (EAE)

**DOI:** 10.3390/cells11223582

**Published:** 2022-11-12

**Authors:** Dimitra Dafou, Eirini Kanata, Spyros Pettas, Nikolaos Bekas, Athanasios Dimitriadis, Garyfalia Kempapidou, Roza Lagoudaki, Paschalis Theotokis, Olga Touloumi, Nikoleta Delivanoglou, Evangelia Kesidou, Konstantinos Xanthopoulos, Nikolaos Grigoriadis, Fotini Nina Papavasiliou, Theodoros Sklaviadis

**Affiliations:** 1 Department of Genetics, Development, and Molecular Biology, School of Biology, Aristotle University of Thessaloniki, 541 24 Thessaloniki, Greece; 2 Neurodegenerative Diseases Research Group, Department of Pharmacy, School of Health Sciences, Aristotle University of Thessaloniki, 541 24 Thessaloniki, Greece; 3 Multiple Sclerosis Center, B’ Neurological Department AHEPA Hospital Thessaloniki, Aristotle University of Thessaloniki, 546 36 Thessaloniki, Greece; 4 Laboratory of Pharmacology, Department of Pharmacy, School of Health Sciences, Aristotle University of Thessaloniki, 541 24 Thessaloniki, Greece; 5 Institute of Applied Biosciences, Centre for Research and Technology Hellas, 603 61 Thessaloniki, Greece; 6 Immune Diversity (D150) Deutsches Krebsforschungszentrum Im Neuenheimer Feld, 280 69120 Heidelberg, Germany

**Keywords:** RNA editing, multiple sclerosis, experimental autoimmune encephalomyelitis, RNA-sequencing, gene expression, microglia, neurodegeneration, neuroinflammation

## Abstract

RNA editing is an epitranscriptomic modification, leading to targeted changes in RNA transcripts. It is mediated by the action of ADAR (adenosine deaminases acting on double-stranded (ds) RNA and APOBEC (apolipoprotein B mRNA editing enzyme catalytic polypeptide-like) deaminases and appears to play a major role in the pathogenesis of many diseases. Here, we assessed its role in experimental autoimmune encephalomyelitis (EAE), a widely used non-clinical model of autoimmune inflammatory diseases of the central nervous system (CNS), which resembles many aspects of human multiple sclerosis (MS). We have analyzed in silico data from microglia isolated at different timepoints through disease progression to identify the global editing events and validated the selected targets in murine tissue samples. To further evaluate the functional role of RNA editing, we induced EAE in transgenic animals lacking expression of APOBEC-1. We found that RNA-editing events, mediated by the APOBEC and ADAR deaminases, are significantly reduced throughout the course of disease, possibly affecting the protein expression necessary for normal neurological function. Moreover, the severity of the EAE model was significantly higher in APOBEC-1 knock-out mice, compared to wild-type controls. Our results implicate regulatory epitranscriptomic mechanisms in EAE pathogenesis that could be extrapolated to MS and other neurodegenerative disorders (NDs) with common clinical and molecular features.

## 1. Introduction

Multiple sclerosis (MS) is a chronic, immune-mediated demyelinating disease of the CNS, which affects more than 2.8 million people worldwide [1]. It is characterized by inflammatory infiltrates, demyelinating plaques, and axonal damage [2]. Among the causes of the origin of multiple sclerosis is the interplay of a person’s genotype with environmental influences [3]. Nevertheless, neither discovery suggests that this applies to the majority of the cases [3]. Patterns of converging features across neurodegenerative diseases (NDs) have been observed [4], calling for a better understanding of the relationships between NDs to reveal disease-specific mechanisms, as well as potentially shared manifestations.

Although diverse disease mechanisms have been implicated in neurodegeneration, a common theme of altered RNA processing has emerged as a unifying contributing factor to NDs [5]. By introducing changes in various classes of RNA, including protein-coding and non-coding RNAs, microRNAs (miRNAs), and circular RNAs (circRNAs), in relation to the relevant genome-encoded information, RNA editing represents an epigenetic process that promotes transcriptome diversity (RNA:DNA differences, RDDs) with potential functional effects [6,7]. Adenosine (A) to inosine (I) or cytidine (C) to uridine (U) chemical alterations (deamination), which result in A-I or C-U conversions, represent the most common type of RNA editing in mammals [5]. The ADAR and APOBEC enzyme families catalyze this nuclear and cytoplasmic co- and/or post-transcriptional process [8]. The ADARs family consists of three members, two of which (ADAR1 and ADAR2) have A-I catalytic action and one of which (ADAR3) acts in a regulatory capacity [9]. On the other hand, the APOBEC family consists of 11 core gene products and their alternatively spliced variants, including the APOBEC-1, activation-induced deaminase (AID), APOBEC-2, APOBEC-3A-H, and APOBEC-4 proteins, with APOBEC-1 being the dominant catalytic enzyme [10].

Given that the brain is one of the tissues in which RNA editing occurs frequently [11], there is an increased interest in research into this post-transcriptional mechanism and its potential role in a number of neurological and neurodegenerative diseases, including MS [12], epilepsy [13], autism [14], amyotrophic lateral sclerosis (ALS) [15], Huntington’s disease (HD) [16], Alzheimer’s disease (AD) [17,18], and Prion diseases [19]. Collectively, it is shown that RNA editing by ADARs and APOBECs plays a crucial role in preserving the balance between healthy and pathological brain functions.

Genetic inactivation of RNA editing enzymes leads to dysregulation and neurodegeneration, characterized by increased inflammation and microglial dysfunction [20,21]. Suppression of cellular dsRNA-mediated innate immune interferon responses by RNA editing has been demonstrated [22,23,24,25,26,27], and this represents a significant potential mechanism behind genetic variations linked to prevalent inflammatory disorders. The absence of RNA editing results in subsequent interferon responses and inflammation [23,24,25]. Aicardi–Goutieres syndrome, a rare autoimmune disease, has been linked to ADAR1 loss-of-function in humans [22], further proving that RNA editing underlies immunological diseases. In addition, a recent study identified RNA editing quantitative trait loci (QTLs), which were significantly enriched in genome-wide association study signals for autoimmune and immune-mediated diseases, thus providing additional information on a role of RNA editing in inflammatory diseases [28]. However, additional research is required, in order to gain experimental functional characterization that will allow for a better understanding of RNA editing in the modulation of inflammation.

RNA editing has been proven to play an important role in inflammation [29,30] and neuroinflammation [20,28]. In recent years, studies focused on its role in immunological control, as well as in its contribution to microglia activation [20,21]. Data revealed that RNA editing is one of the main mechanisms that facilitates microglia plasticity and activation [8]. Microglia serve as the main and most important source of immune response in the CNS. Lately, it has been proven that they represent an extremely heterogenous population with high plasticity and play an important role in NDs [31], including experimental autoimmune encephalomyelitis (EAE), one of the most commonly used mouse models for studying MS [32]. Microglia change their transcriptional profile in MS and EAE, and in contrast to other neurodegenerative disorders, EAE microglia exhibit a prominent inflammatory phenotype [33]. Microglia activate many effector processes, including the release of pro- and anti-inflammatory mediators, phagocytosis, synaptic pruning, and antigen presentation, that might be neuroprotective or harmful in MS and EAE [34]. While microglia’s first activation, which aims to resolve the injury, is beneficial, persistent stimulation leads to neurodegeneration [35].

Herein, we describe a screen for transcriptome-wide, microglia-related RNA editing in EAE. The EAE model has been extensively used in research as an animal model of MS, primarily in C57BL/6 mice immunized with myelin oligodendrocyte glycoprotein (MOG) peptide 35–55 (MOG_35–55_). In EAE and MS, microglia have been found inside demyelinated lesions, with substantial numbers residing on the leading edge, suggesting that they may play an active role in the demyelination process [35]. We chose to study the effects of the loss of RNA editing during EAE induction in C57BL/6 APOBEC-1 knock-out (KO) mice, a mouse model that previously highlighted the vital role of APOBEC-1-mediated RNA editing in keeping the balance between microglia’s homeostatic and activated immunological functions [20]. Conditional ADAR KO would be a more complicated model to handle and is included in our future plans to be explored. We observed that loss of RNA editing leads to a severe immune response, with clinical earlier signs than wild-type (WT) mice, and significantly higher microglia density and gliosis, exacerbating disease pathophysiological features, thus concluding that RNA editing confers a protective role during ΕAΕ disease progression. Our results indicate epitranscriptome variation, especially at the clinical disease stages of MOG_35–55_-induced EAE pathology, which could lead to the elucidation of contributing mechanisms (i.e., lysosomal, endoplasmic reticulum stress processes) and identification of potential therapeutic strategies.

## 2. Materials and Methods

### 2.1. RNA Editing Analysis

RNA editing analysis was performed on publicly available RNA-seq data (15–20 million reads per sample, GEO accession number GSE59725) from brain and spinal cord microglia isolated from non-EAE (naïve) or EAE female C57BL/6 mice. Disease was induced by subcutaneous MOG_35–55_ administration, and samples were collected at pre-clinical (7 days post-induction, no clinical signs) and acute (14 days post-induction, clinical score 3) disease stages. Data correspond to microglia pools from 10 animals per group [36]. Raw RNA-seq data quality control, pre-processing (including adapter, 5′and 3′ trimming to minimize false positive RDDs identification due to random hexamer primer bias), and mapping to the murine reference genome (mm10) were performed as previously described [19]. Table S1 summarizes quality control and alignment data per sample. The following thresholds were used: (a) base quality: 25, (b) read depth: 10, (c) bases supporting the variation: 3, (d) editing frequency: 0.1, (e) *p*-value (for FDR and Fisher’s exact test for REDItools and VarScan analysis, respectively): 0.05. Multimapping reads and reported SNPs (based on dbSNP (v.142)) were excluded from the analysis. Custom *R* scripts were used for further statistical analysis for extracting, processing, and visualizing C-U and A-I RNA editing events (Figure S1). The GitHub repository containing the in-house scripts used can be accessed (https://github.com/athanadd/RNA-editing-in-EAE (accessed on 11 September 2017)).

### 2.2. Pathway Analysis

In order to identify processes affected by RNA editing, edited transcripts were subjected to pathway analysis using the Enrichr software (https://amp.pharm.mssm.edu/Enrichr/ (accessed on 20 September 2017)) [37,38]. The mouse Kyoto Encyclopedia of Genes and Genomes (KEGG, 2019 release) was used. Enrichment scores for RNA-edited transcripts were determined per condition (naïve, pre-clinical, acute disease), based on corresponding *p*-values, expressed as (-log(*p*-values)) and plotted. Clustergrams visualizing the involvement of edited targets (up to sixty) in the top ten enriched terms per condition were generated based on *p*-values and extracted from the Enrichr software.

### 2.3. RNA Editing Experimental Validations

Experimental validations of RNA editing alterations entailed one ADAR-mediated A-I-edited (*Mpeg1*) and one APOBEC-mediated C-U-edited (*B2m*) disease-associated target, predicted to display both differential editing and differential gene expression from our transcriptomic and RNA editing analysis. RNA editing verification analysis was performed using both murine brain-isolated microglia and brain tissue material.

#### 2.3.1. Microglia Isolation

Microglia were isolated from the brain of naïve and EAE female C57BL/6 mice, following a gradient Percoll method. Briefly, brains were excised from animals sacrificed by cervical dislocation, following ether anesthetization. Brain tissue was minced and homogenized in 1x Hank’s Balanced Salt Solution (HBSS, 14025092, Gibco) on ice and enzymatically dissociated with 1 mL Accutase (SCR005, EDM, Millipore) and 1.25 μL of 200 U/mL DNAseI (DN25-1G, Sigma) in a stirring incubator (Eppendorf, 37 °C, 40 min, 175 Hz), followed by a short incubation (37 °C, 5 min) in the presence of 20 μL 0.5 M EDTA. Dissociated cells were pelleted (453× *g*, 10 min, 20 °C), resuspended in 10 mL 70% Percoll (P1644, Sigma), overlayed with 10 mL 35% Percoll, followed by 10 mL 1xPBS, and centrifuged (1811× *g*, 45 min, 20 °C, no brake). The microglia containing layer, occurring below the myelin dense white ring, was transferred into a clean tube, supplemented with 1× PBS up to 40 mL, and centrifuged (805× *g*, 10 min, RT) to pellet the cells. The cell pellet was resuspended in 100 μL RNAlater (76106, Qiagen) and stored at −20 °C until further processing for subsequent RNA extraction.

#### 2.3.2. Microglia RNA Extraction and Reverse Transcription

Total RNA from microglia was extracted with the RNeasy Mini kit (74104, Qiagen), according to the manufacturer’s instruction, and eluted in 35 μL RNase-free water. The quantity and purity of the preparations were spectrophotometrically assessed (NanoDrop™ 2000/2000c Spectrophotometer). cDNA was synthesized with the PrimeScript™ RT reagent Kit (RR037A, TAKARA) following the manufacturer’s instructions. Amplification of the commonly used CD11b microglia marker (primers used CD11b-F: AAGGATTCAGCAAGCCAGAA, CD11b-R: GGAGGGATGAGAGTCCACAT, annealing at 60 °C) was performed on acquired cDNAs to assess the effectiveness of microglia isolation.

#### 2.3.3. Brain Tissue RNA Extraction and Reverse Transcription

RNA extracted (Trizol (15596018, Invitrogen) method, as per manufacturer’s instructions) from brain tissue of naïve (*n* = 3, bulk tissue (whole brain and cortex)) and acute EAE (*n* = 3, bulk tissue (whole brain and cortex)) mice was used for RNA editing validations. cDNA was synthesized with the PrimeScript™ RT reagent Kit (RR037A, TAKARA) using 500 ng RNA in 10 μL reactions, including both the OligodT primers and random 6mers, according to the manufacturer’s instructions, and used for subsequent editing validations.

#### 2.3.4. Genomic DNA Isolation

Genomic DNA was extracted from spleen or lymph node tissue isolated from the same naïve and EAE animals for which RNA analyses were performed, using the spin column PureLink™ Genomic DNA Mini Kit (K1820-01, Invitrogen), according to the manufacturer’s instructions. Column-bound DNA was eluted in two individual steps, using 50 μL elution buffer per elution.

#### 2.3.5. RNA Editing Validations

Target transcript fragments, including the positions of interest, were amplified using 10 ng of the prepared cDNAs as template and the high fidelity Q5 DNA polymerase (M0491L, NEB), according to the manufacturer’s instructions. The following primer sets and annealing temperatures were used: mo-*Mpeg1*-3′UTR-F: TGGCAGATCAAGCAGTATGG, mo-*Mpeg1*-3′UTR-R: TTAACGCTCAAGAGGCAGGT (67 °C), mo-*B2m*-3′UTR-F: CTGACCGGCCTGTATGCTAT, mo-*B2m*-3′UTR-R: TGGGGGTGAGAATTGCTAAG (68 °C). Amplification products (773 bp for *Mpeg1*, 717 bp for *B2m*) were purified using the PCR clean-up and gel extraction kit (740609.250, Macherey-Nagel) and were either directly sequenced (sequencing primers, same as the corresponding amplification primers) or subjected to TA cloning before sequencing analysis. Cloning was applied in all cases where brain tissue material was used, to compensate for lower editing rates expected to be observed due to dilution of the microglia-enriched editing events identified by our in silico analysis. The same procedure was also used for the analysis of editing events in the *B2m* transcript from both microglia and whole brain tissue, to enable identification and precise quantification of low frequency (<20%) editing events predicted in this target. Direct Sanger sequencing was preferred for the analysis of microglia *Mpeg1* amplification products, for which a clear difference in editing frequencies between naïve and acute EAE and higher editing rates were predicted.

For TA cloning, the pDrive vector (Qiagen, PCR cloning kit, Cat No. 231124) was used. PCR products subjected to an additional A-tailing step were further cleaned-up and ligated in the pDrive vector using a 20× excess molar ratio of insert, relative to the vector. Plasmids corresponding to at least 30 clones isolated from transformed TOP10 *E. coli* cells were prepared using the Nucleospin plasmid kit (740588.250, Macherey-Nagel) and sequenced in both directions (sequencing primers: M13F: TGTAAAACGACGGCCAGT, M13R: CAGGAAACAGCTATGACC). Sequencing analysis was also performed on similarly amplified and PCR cleaned-up *Mpeg1* and *B2m* containing gDNA (sequencing primers, same as the corresponding amplification primers). All sequencing reactions were performed at Cemia (Larisa, Greece) using the v3.1 BigDye Terminator chemistry (ABI) and were analyzed on a 3730 genetic analyzer (ABI). Raw sequencing data were processed with the Bioedit software v7.09.0. RDDs corresponding to RNA editing events were identified through alignment of cDNA sequencing data to the corresponding gDNA sequence.

### 2.4. Gene Expression Validations

Differential expression of the *Mpeg1* and *B2m* transcripts between naïve and acute EAE microglia was experimentally verified by quantitative real-time PCR (qRT-PCR, SYBR Fast Universal 2X qPCR Master Mix Kit, KK4601, Kapa Biosystems), employing the 2^-ΔΔCt^ method and using *Gapdh* for data normalization. The following primer sets were used: mo-q*Mpeg1*-F: TGGCAGATCAAGCAGTATGG, mo-q*Mpeg1*-R: CTCACTGTGACTTGCGCATT, mo-q*B2m*-F: GCCGAACATACTGAACTGCT, mo-q*B2m*-R: CTTGATCACATGTCTCGATCCC, mo-GAPDH-F: ACTCCACTCACGGCAAATTC, mo-GAPDH-R: TCTCCATGGTGGTGAAGACA. Reactions were performed using 10 ng cDNA as template and 0.7 μM of each primer in a final volume of 14 μL. Thermal cycling and data acquisition were performed in a 7500 Fast Real-Time PCR System (Applied Biosystems), under the following conditions: initial denaturation at 95 °C for 2 min, 40 cycles consisting of denaturation for 5 s at 95 °C, annealing at 60 °C for 20 s, and extension at 72 °C for 20 s. All reactions were set in triplicates.

### 2.5. In Vivo Study

#### 2.5.1. Animals Used for The In Vivo Study

All animal experiments were performed in accordance with national and institutional requirements, under the supervision of the regional veterinary services, which reviewed and approved them. Animals were housed in the animal facility of the B’ Neurological Department, AHEPA University Hospital (accreditation number EL 54 BIO 29), and the experimental protocol was approved by the IACUC and the local veterinary services.

APOBEC-1^+/−^ C57Bl/6 mice [39] were kindly provided by Prof. Fotini Papavasiliou (DKFZ). To confirm the genotype, gDNA was extracted from ear lobe tissue, using phenol:chloroform:isoamyl alcohol. Wild-type (600 bp) and APOBEC-1 KO (250 bp) fragments were identified through two amplification reactions using appropriate forward primers (Apobec1-WT-F: GACTATCCAGATCATGACAGAGC, Apobec1-KO-F: GGCCAGCTCATTCCTCCACTCATGATC) and a reserve primer (Apobec1-R: CCACAGATGGGGGTACCTTGGCCAATAAG) at 58 °C annealing temperature.

#### 2.5.2. EAE Induction

EAE was induced in wild-type (*n* = 9) and APOBEC-1 KO (*n* = 11) 8-to-10 weeks old female C57BL/6 mice through subcutaneous injection into the right para-lumbar region of 50 μg myelin oligodendrocyte glycoprotein 35–55 peptide (MOG_35–55_; kindly provided by John Matsoukas, Department of Chemistry, University of Patras) emulsified in 100 μL complete Freund’s adjuvant (CFA, F5506, Sigma) containing 4 mg/mL desiccated *Mycobacterium tuberculosis* H37Ra (231141, Difco Laboratories). Immediately thereafter, and on day 2, mice were inoculated intraperitoneally with 400 ng of reconstituted lyophilized pertussis toxin from *Bordetella pertussis* (P2980, Sigma) in 500 μL of filtered 1x PBS. An additional injection of MOG_35–55_ peptide in CFA was delivered 7 days later (MOG booster) into the left para-lumbar region to induce severe EAE. Clinical scores were observed and recorded daily; clinical score 0: no symptoms, clinical score 1: limp tail, clinical score 2: limp tail and inability to right itself when placed on its back, clinical score 3: one limb plagic, score 4: paraplegia with forelimb weakness, score 5: quadriplegia or moribund [40,41] Clinical disease scores (mean ± SE) were plotted relative to time for each group. Mean maximal score (mMS), mean area under the curve (mAUC), and mean day of disease onset (dDO) between the two groups were compared through statistical analysis [41]. All animals were sacrificed at 21 days post-disease induction (acute disease stage), through anaesthesia and intracardial perfusion, followed by 4% *v*/*v* paraformaldehyde fixation. Brain and spinal cord tissues were harvested, fixed in fresh 4% *v*/*v* paraformaldehyde for 24 h, and further processed for paraffin embedment. Paraffin-embedded tissues were sectioned using a microtome, and consecutive sections of 6 μm thickness were cut for subsequent staining.

#### 2.5.3. Tissue Staining

Hematoxylin and eosin (HE) and luxol fast blue (LFB) staining were performed following standard staining protocols. EAE lesions were evaluated on digital images collected from spinal cord sections and processed using the ImageJ software, https://imagej.nih.gov/ij/, (accessed on 20 September 2017). The percentage of inflammatory cell infiltration was assessed through analysis of HE-stained sections at 40× magnification. The demyelination percentage on white matter was assessed through analysis of LFB-stained sections at 2.5× magnification.

#### 2.5.4. Immunohistochemistry

Spinal cord sections were subjected to immunohistochemical analysis, as previously described, to assess gliosis (Iba1), astrocytosis (GFAP), and T cell infiltration (CD3) [42]. The size and percentage of the total area covered by stained cells was calculated using the ImageJ software and pictures captured at 40× magnification.

### 2.6. Statistical Analysis

Data are presented as mean ± standard error, unless otherwise indicated. For statistical analysis, the GraphPad prism software was used (version 7.0 and 8.0). The tests used in each case are indicated when pertinent results are presented. Statistical significance is indicated as follows: * *p* ≤ 0.05, ** *p*≤ 0.01, *** *p* ≤ 0.001.

## 3. Results

### 3.1. Microglia Editomes during EAE Progression

Recent reports provide accumulating evidence on the involvement of RNA editing alterations in brain disorders, including neurodegenerative [18,19,43], neurological [13,14,44], and autoimmune diseases [45,46,47]. Extending our previous research in this field, the present study focused on EAE, as a representative of demyelinating disorders entailing autoimmune responses, inflammation, and degeneration.

Utilizing next-generation RNA sequencing data corresponding to microglia during EAE progression (GSE GSE59725) [36] and our previously developed in-house RNA editing analysis pipeline, we established global RNA editing profiles (*editomes*) of murine microglia during disease progression. To increase accuracy and limit false positive RDDs, an inherent pitfall of RNA editing analysis approaches, we combined different analysis suites and introduced filtering in critical processing steps. We excluded reads mapping to multiple genomic loci, as well as known pseudogenes and reported SNPs. Furthermore, we applied stringent criteria considering base quality, read depth, and statistical analysis (FDR and Fischer’s exact test *p*-value) for the identification of high confidence RNA editing events (Figure S1). In addition, as a means of quality control of our analyses, we determined the distribution of the 12 types of RDDs detected in the studied murine microglia dataset (Figure S2).

**Figure 1:**
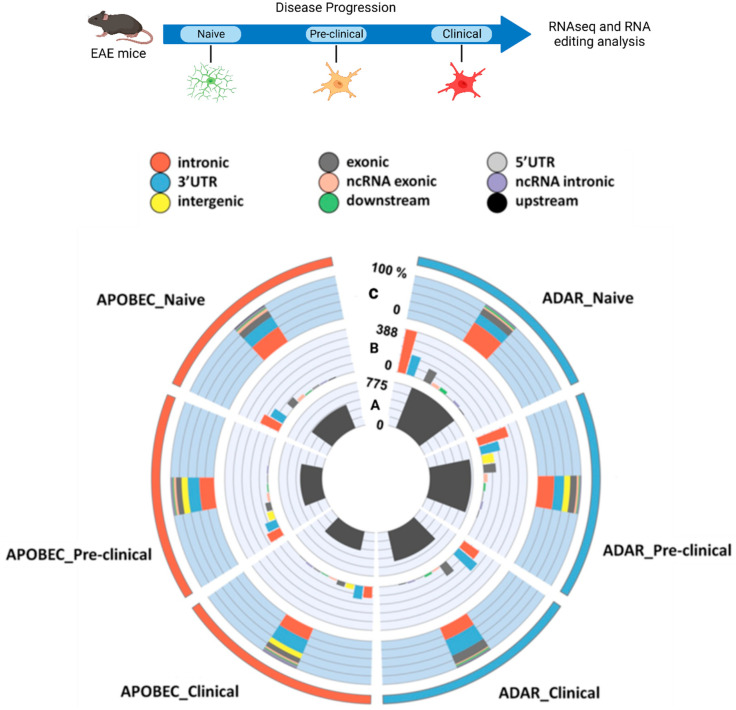
RNA editing is reduced in murine microglia and shifts towards 3′UTR in clinical disease. Graphic representation of murine microglia RNA editomes, following EAE induction. Presented data are based on meta-analysis of previously published RNA-seq data (GSE59725, [36]), following an in-house developed pipeline for RNA editing events identification. Ring A: Absolute number of RNA editing events identified per editing-mediating enzyme and time-point (naïve, pre-clinical, clinical). Both ADAR- and APOBEC-mediated editing were reduced as disease progressed (for detailed numbers, please also see Tables S2–S4). Ring B: Genomic distribution (color code legend on top of the graph) of RNA editing events (absolute numbers) for each RNA-editing mediating enzyme and time-point. A shift towards increased 3′UTR editing was observed as disease progressed. Ring C: Percent genomic distribution of editing events across functional gene regions (color code legend on top of the graph). For a detailed list of editing events identified per editing enzyme and time-point, please refer to Supplementary Files S1 and S2. Top panel figure created in Biorender.com (accessed on 9 September 2022).

Detailed lists of high confidence ADAR (A-I) and APOBEC (C-U)-mediated RNA editing events identified in naïve, pre-clinical, and acute EAE murine microglia are provided in Supplementary Files S1 and S2. Figure 1 summarizes microglia editomes (absolute numbers and percentages) in naïve and EAE microglia during disease progression. Tables S2–S4 present detailed pertinent data.

We observed a progressive reduction of global editing during disease progression (naïve: 1225, pre-clinical: 1134, acute: 891), corresponding to 7% and 27% reductions at the pre-clinical and clinical disease stages, respectively, relative to the naïve condition (Table S2). The observed reduction is mainly attributed to APOBEC at the pre-clinical disease stage (4% and 13% reductions of ADAR- and APOBEC-mediated editing, compared to naïve A-I and C-U editing), while a slight overrepresentation of ADAR editing reduction was found at the clinical stage (30% reduction for A-I, 23% reduction for C-U editing, Tables S3 and S4).

Considering naïve microglia, A-I changes (775) were almost two-fold over-represented, compared to C-U (450) (Figure 1, Ring A, Tables S3 and S4), further verifying the previously reported abundance of A-I editing. In naïve microglia, most editing events were identified in non-coding regions (intronic: 46.4%, 3′UTR: 25.2%, ncRNA-exonic: 4.5%, ncRNA-intronic: 2.5%, upstream/downstream: 4.2%, 5′UTR: 1.6%), while exon-occurring editing represented the 15.4% of total editing (Table S2). This genomic distribution pattern was equally contributed by both editing enzyme families, as similar distribution patterns were detected when ADAR- and APOBEC-mediated editing were considered separately (Tables S3 and S4, Figure 1. Ring B, C).

We noticed altered genomic distribution of RNA editing events as diseases progressed, characterized by a shift towards increased 3′UTR (25.2% naïve, 24.8% pre-clinical, 37.5% clinical) and intergenic editing (0%: naïve, 14.2%: pre-clinical, 4.7%: clinical), as opposed to decreased intronic editing (46.4%: naïve, 36.0%: pre-clinical, 29.7%: clinical) (Table S2). Alterations in 3′UTR and intronic editing representation were almost equally contributed by both ADARs and APOBECs (Tables S3 and S4, Figure 1, Ring B, C), while increases in RNA editing events in intergenic regions were attributed solely to APOBEC-mediated editing alterations (Tables S3 and S4, Figure 1, Ring B, C). Interestingly, we also noticed a trend towards enrichment of exonic A-I editing at the clinical disease stage (15.4% in naïve microglia versus 20.3% in clinical disease, Table S3, Figure 1, Ring B, C).

**Figure 2:**
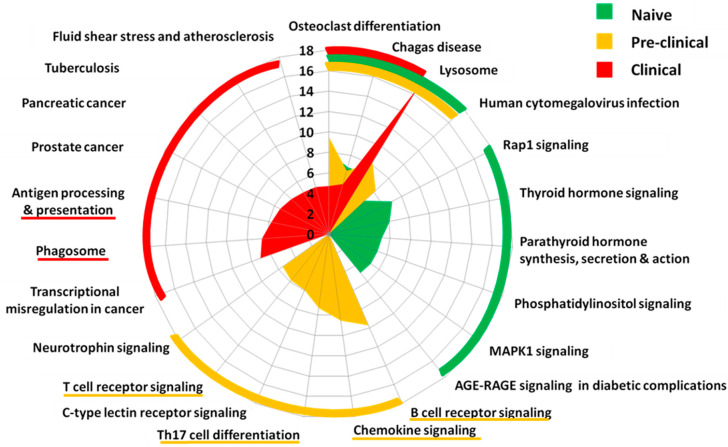
RNA editing occurs at microglial transcripts involved in differential signaling under normal (naïve) and EAE (pre-clinical, clinical) conditions. Visualization of pathway analysis (KEGG 2019 mouse database, through Enrichr, https://amp.pharm.mssm.edu/Enrichr/ (accessed on 20 September 2017)) performed on edited targets at each time-point. The graph provides the enrichment (-log10(*p*-value)) of the top 10 pathways identified per condition (ranked based on *p*-value). Three pathways (‘Osteoclast differentiation’, ‘Chagas disease’, ‘Lysosome’) are commonly identified in all three conditions. Among these, the pathway ‘Lysosome’ is over-represented in clinical disease. Microglia response signaling pathways are enriched in pre-clinical disease, while in clinical disease, the pathways ‘Antigen processing and presentation’ and ‘Phagosome’ are also enriched. For detailed illustrations of the 60 most enriched RNA-edited transcripts in corresponding pathways and time-points, refer to Figures S3–S5. Editing events occurring at intergenic regions were not included in this pathway analysis.

Pathway analysis revealed that transcripts undergoing RNA editing in naïve microglia are involved in the signal transduction processes (phosphatidylinositol signaling system, mmu04070) associated with cellular growth, development, and metabolism (thyroid hormone signaling pathway, mmu04919), cell–cell and cell–matrix interactions and cell polarity (Rap1 signaling pathway, mmu04015), cell proliferation, differentiation, and migration (MAPK signaling pathway, mmu04010), calcium and phosphorus homeostasis (parathyroid hormone synthesis, secretion, and action pathway, mmu04928), immune receptor-related processes (AGE-RAGE signaling pathway in diabetic complications, mmu04933, osteoclast differentiation, mmu04380), immune responses to infection (Chagas disease, mmu05142, cytomegalovirus infection, mmu05163), and cellular processes related to lysosome function (lysosome, mmu04142). These data suggest a role of RNA editing in orchestrating diverse cellular and inter-cellular processes in non-disease conditions (Figure 2). Interestingly, pre-clinical disease microglia editome analysis highlighted further enrichment in the immune response signaling pathways (B cell receptor signaling pathway (mmu04662), chemokine signaling pathway (mmu04062), T cell receptor signaling pathway (mmu04660), Th17 cell differentiation (mmu04659), and C-type lectin receptor signaling pathway (mmu04625)), indicating the potential functional effects of the observed RNA editing alterations on microglia activation and function at early disease stages (Figure 2). Moreover, additional immune response pathways (antigen processing and presentation, tuberculosis) and pathways entailing oxidative and inflammatory conditions (e.g., fluid shear stress and atherosclerosis) were enriched in acute EAE microglia editomes. Further, an outstanding enrichment of edited transcripts within the lysosome pathway was observed. Additionally, the phagosome pathway was enriched in acute EAE microglia editomes. These results suggest further functional roles of RNA editing, highlighting its involvement in cellular clearance pathways in acute EAE microglia (Figure 2). In order to provide a means of target ranking and identifying edited transcripts with potential functional roles in EAE, we utilized clustergrams depicting the involvement of the top 60 edited transcripts in the top ten enriched pathways (ranking based on *p*-values) in naïve, pre-clinical, and acute EAE microglia editomes (Figures S3–S5).

### 3.2. Experimental Verification of RNA Editing Alterations

As a proof-of-concept of our in silico study highlighting RNA editing alterations in EAE, we proceeded to the experimental validation of the selected A-I and C-U RNA editing events, utilizing the affected tissue material from naïve and acute EAE mice. The *Mpeg1* (A-I) and *B2m* (C-U) transcripts were selected as representatives of ADAR and APOBEC targets, respectively. These targets were selected because (a) both *Mpeg1* and *B2m* display RNA editing and gene expression differences between naïve and acute EAE microglia, as determined by our in silico editing analysis and further experimental verification (Figure S6) of previously reported gene expression analysis [36] (Table S1), respectively, and (b) both targets have been functionally implicated in EAE progression [48,49]. Moreover, *B2m* has been highlighted by our pathway analysis (Figures S3–S5).

We focused on the verification of 3′UTR-residing RNA editing events, due to the high prevalence of 3′UTR editing and to previous knowledge associating 3′UTRs with gene expression modulation. Both brain microglia and brain tissue from naïve and acute EAE mice were used for editing validations.

**Figure 3:**
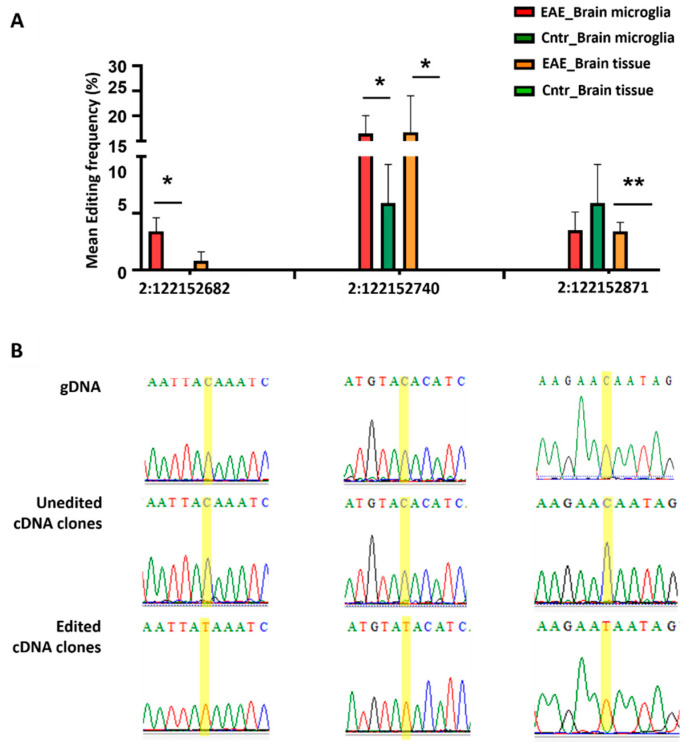
Naïve and acute EAE murine microglia displayed different profiles and frequencies of APOBEC-mediated (C-U-T) RNA editing within the 3′UTR of the *B2m* transcript (increased editing frequency in disease condition); a similar *B2m*-3′UTR editing pattern was detected in brain tissue. (**A**)**.** Graphical representation of the experimentally validated and statistically significant C-U-T editing events between naïve and acute EAE microglia and brain tissue, based on the direct comparison of cDNA clones with corresponding gDNA sequences through Sanger sequencing. Four technical replicates of brain microglia samples were used per condition (EAE, Cntr), including at least 20 (EAE) or 10 (Cntr) clones per replicate. Brain tissue analysis was performed on three biological replicates per condition (*n* = 3 EAE, *n*= 3 Cntr), including the analysis of at least 30 clones per sample. For editing frequency quantification, the percentage (%) of cDNA clones displaying the edited base relative to the total number of tested clones per sample was utilized. The bars indicate the mean editing frequency per sample type and condition. Error bars correspond to standard error. For Statistical analysis, one-tailed unpaired t-test was performed. Significance symbols are as follows: * *p* ≤ 0.05, ** *p*≤ 0.01. (**B**)**.** Representative chromatograms from Sanger sequencing analysis of gDNA and un-edited and edited cDNA clones harboring the positions of interest (highlighted in yellow). Detailed Sanger sequencing analysis data (alignments) is provided in Figure S7 (microglia) and Figure S8 (brain tissue).

Regarding *B2m* analysis, we identified several low frequency 3′UTR-residing C-U editing events and observed a shift towards increased number of edited sites in EAE, compared to the naïve condition, consistent with our in silico analysis. Specifically, through direct comparison of cDNAs with corresponding gDNAs, we experimentally verified C-U editing in three out of six of the predicted positions in naïve microglia (2: 122,152,740 identified in 2/4 replicates, 2: 122,152,742 identified in 1/4 replicates, and 2: 122152871, identified in 2/4 replicates) and in nine out of nineteen predicted positions in acute EAE microglia (2: 122152682, identified in 3/4 replicates, 2: 122152687, identified in 1/4 replicates, 2: 122152707, identified in 1/4 replicates 2: 122152740, identified in 4/4 replicates, 2: 122152742, identified in 3/4 replicates, 2: 122152751, identified in 1/4 replicates, 2: 122152871, identified in 3/4 replicates, 2: 122152912, identified in 1/4 replicates, 2: 122152928, identified in 1/4 replicates). Editing at positions 2: 122,152,682 and 2: 122,152,740 showed statistically significant differences between naïve and acute EAE microglia (one tailed, unpaired t-test, *p*: 0.0133 and *p*: 0.0429, respectively, Figure 3, Table S5, Figure S7).

A similar editing pattern was observed when *B2m* 3′UTR analysis was performed in brain tissue from naïve and acute EAE mice, allowing for the identification of three edited sites in the naïve condition (2: 122152687, 2: 122152707, 2: 122152787, all of which were detected in 1/3 tested samples), in contrast to the eleven edited sites in acute EAE (2: 122,152,682 detected in 1/3 samples, 2: 122,152,694 detected in 1/3 samples, 2: 122,152,740 detected in all 3 tested samples, 2: 122,152,742 detected in 1/3 samples, 2: 122,152,745 detected in 1/3 samples, 2:122152751 detected in 1/3 samples, 2: 122,152,759 detected in 1/3 samples, 2: 122,152,763 detected in 2/3 samples, 2: 122,152,845 detected in 1/3 samples, 2: 122,152,871 detected in all three tested samples, and 2: 122,152,928 detected in 2/3 tested samples). Similar to the microglia analysis, C-U editing rates at position 2:122152740 were statistically significant different between naïve and acute EAE brain tissue samples (p: 0.0403, Figure 3, Table S5, Figures S7 and S8). Editing at position 2: 122,152,682 did not emerge as statistically significant in brain tissue analysis; however, another site (2: 122152871) was identified as significant (p: 0.0063) (Figure 3, Table S5, Figures S7 and S8). Please note that, due to the identification of gDNA variation in at least one of the tested samples, C-T changes identified at positions 2: 122152787, 2: 122,152,826, and 2: 122,152,902 were not reported as experimentally verified RNA editing events. Similarly, position 2: 122,152,804 was not reported as edited, as it had been previously shown to harbor an SNP (rs13467842).

**Figure 4:**
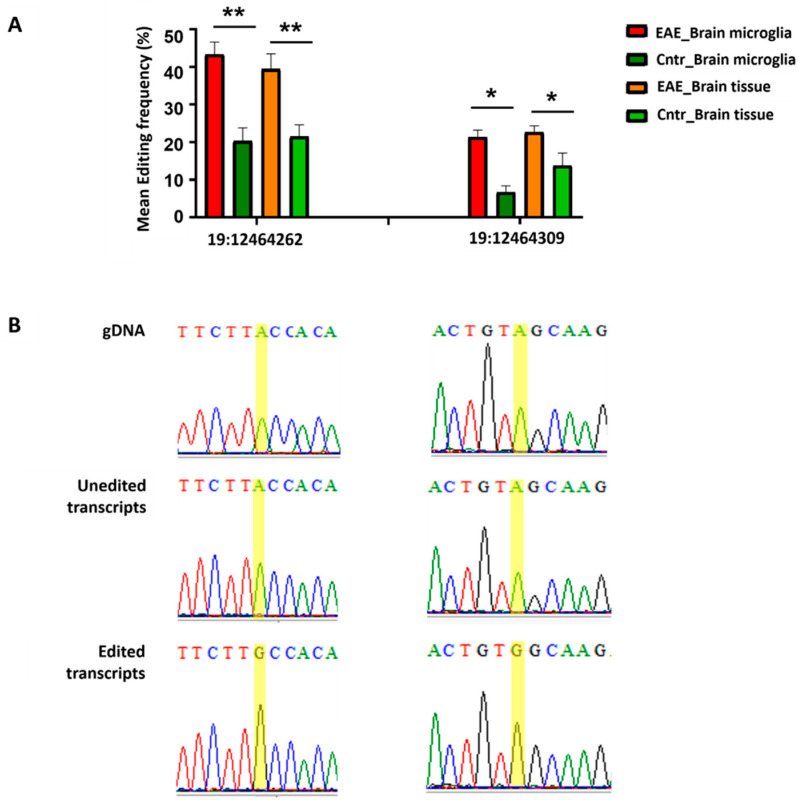
Naïve and acute EAE murine microglia displayed different frequencies of ADAR-mediated (A-I-G) RNA editing within the 3′UTR of the *Mpeg1* transcript (increased editing frequency in disease condition); a similar *Mpeg1*-3′UTR differential editing frequency pattern was detected in brain tissue. (**A**). Experimental validation of A-I-G editing events, based on the direct comparison of cDNAs with corresponding gDNA sequences through Sanger sequencing. Three technical replicates of brain microglia samples were used per condition (EAE, Cntr); editing frequencies were calculated from corresponding Sanger sequencing chromatograms of PCR-amplified cDNAs encompassing part of the target 3′UTR flanking the predicted editing sites. ImageJ was used to calculate editing frequencies as the percentage of G peaks, relative to A peaks, per editing site. Brain tissue analysis was performed on three biological replicates per condition (*n* = 3 EAE, *n*= 3 Cntr), including the analysis of at least 30 clones per sample. For editing frequency quantification, the percentage (%) of cDNA clones displaying the edited base, relative to the total number of tested clones, per sample was measured. Bars indicate the mean editing frequency per sample type and condition. Error bars correspond to standard error. For statistical analyses, one-tailed unpaired t-test was performed. Significance symbols are as follows: * *p* ≤ 0.05, ** *p*≤ 0.01. (**B**). Representative chromatograms from Sanger sequencing analysis of gDNA and un-edited and edited transcripts harboring the positions of interest (highlighted in yellow). Detailed Sanger sequencing analysis data (alignments) is provided in Figure S9 (microglia) and Figure S10 (brain tissue).

As far as the ADAR target is concerned, we experimentally validated two sites undergoing A-I editing in both the normal and disease conditions (19: 12464309, 19: 12464262) in the *Mpeg1* 3′UTR and identified increased editing frequencies at these sites in acute EAE, compared to naïve animals. Specifically, we observed a statistically significant increased editing frequency in acute EAE for both the microglia and the brain tissue samples (Figure 4, Table S5, Figures S9 and S10).

### 3.3. In Vivo Study

Aiming to provide in vivo data regarding the contribution of RNA editing in EAE, we utilized wild-type and APOBEC-1-deficient (APOBEC-1 Knock-out, KO) C57BL/6 mice [39]. APOBEC-1 is enriched in microglia in the murine brain [20] and represents the main C-U RNA editing enzyme in mammals. APOBEC-1 KO mice, in contrast to ADAR KOs (which require rescue of crucial editing sites) are viable, without displaying significant phenotypic deviations. EAE was induced in wild-type (*n* = 9) and APOBEC-1 KO (*n* = 11) mice, and clinical signs were scored daily, as described in the Materials and Methods section. Weight loss preceded clinical symptoms occurrence and evolved along with disease progression and severity in both groups, reaching a statistical significance only close to the disease peak (Figure S11).

**Figure 5:**
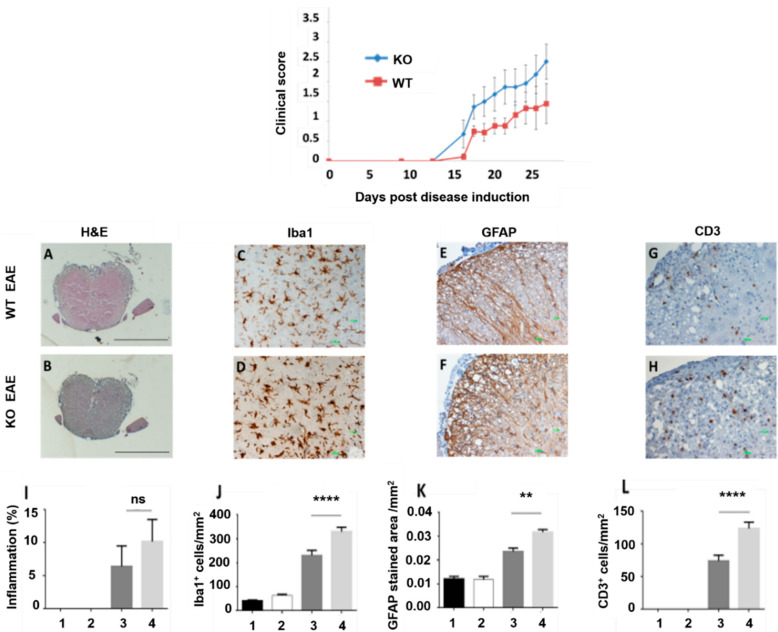
APOBEC-1 KO mice present higher severity EAE, as assessed by clinical disease score and underlying histopathological aspects, compared to wild-type animal (WT)s. Top panel: Mean clinical scores from active EAE with MOG_35−55_ in APOBEC-1 KO and wild-type C57BL/6 mice. Clinical scores were assessed daily, beginning on day 7 and ending on day 21 (acute disease stage). Error bars represent means ± SEM (p< 0.001, two-tailed *t*-test). Low panel: Histochemical analysis of spinal cord sections from MOG_35–55_-immunized mice. (**A**,**B**) H&E (bars represent 1000 um), (**C**,**D**) Iba1, (**E**,**F**) GFAP, and (**G**,**H**) CD3 (bars represent 100 um) staining of WT and APOBEC-1 KO EAE mice. (**I**) Quantification of the percent area of inflammation, based on hematoxylin eosin-stained spinal cord sections of naïve and EAE, WT, and APOBEC-1 KO mice. (**J**–**L**) Quantification of Iba1^+^-stained cells/mm2, GFAP^+^-stained area/mm2, CD3^+^-stained cells/mm2, respectively, on spinal cord sections of naïve and EAE, WT, and APOBEC-1 KO mice. All samples were collected 21 days post-induction. Error bars represent means ± SEM (**** *p* < 0.0001, ** *p* < 0.01, ns = non-significant, Kruskal–Wallis test). 1: wild-type naïve group (*n* = 5), 2: APOBEC-1 KO naïve group (*n* = 5), 3: wild-type acute EAE group (*n* = 9), 4: APOBEC-1 KO acute EAE group (*n* = 11).

Clinical disease occurred earlier in APOBEC-1 mice (approximately at 13 days post-disease induction), compared to wild-type animals, which presented the first symptoms ~1 day later (mean day of disease onset (dDO) (13.63 ± 0.18 vs. 14.50 ± 0.26, respectively; *p* < 0.05). Loss of tail tone and general weakness were the first disease signs. Limb tail and mild-to-severe disabilities, including paralysis of back limbs or both forth and back, were detected in both groups as the disease progressed to its peak (20–21 days post-induction), at which point, all animals were sacrificed. A higher disease severity was observed in APOBEC-1 KO mice, as determined by the statistically significant difference in mean maximal score (mMS) values between the APOBEC-1 KO and wild-type EAE groups (4.68 ± 0.16 and 2.43 ± 0.42, respectively, *p* < 0.01). Similarly, values of the mean area under the curve (mAUC) for APOBEC-1 KO and wild-type EAE animals, corresponding to 12.74 ± 0.26 and 6.77 ± 0.17, respectively, were statistically significant (*p* < 0.05) (Figure 5, top panel). Immunohistochemical analyses performed on spinal cord slides prepared from acute EAE (day 21) APOBEC-1 KO and wild-type mice further indicated more severe disease in the APOBEC-1 KO group. In more detail, we identified a trend towards increased inflammation, assessed by hematoxylin and eosin (H&E) staining (Figure 5, low panel A, B, I), which did not reach statistical significance. However, immunohistochemical analysis for the assessment of EAE hallmarks, including gliosis (Iba1 staining, Figure 5, low panel C, D, J), astrocytosis (GFAP staining, Figure 5, low panel E, F, K), and T cell infiltration (CD3 staining, Figure 5, low panel G, H, L), showed statistically significant increases of these processes in APOBEC-1 KO, compared to wild-type mice.

Collectively, our in vivo study data suggest a ‘critical’ role for APOBEC-1, whose absence is associated with increased EAE severity. Additionally, a significant reduction in total RNA editing events during the course of disease for both ADAR and APOBEC was observed, whereas the individual key immune regulation (*B2m*) and pro-apoptotic immunomodulatory (*Mpeg1*) genes used for validation demonstrated a statistically significant increased number of editing events in the diseased versus control animals.

## 4. Discussion

RNA editing is not essential for viability, provided that crucial A-I edited sites are rescued [50]; however, it is recognized as a significant contributor to transcriptome and proteome diversification in the brain [6,51,52,53], with in vivo brain disease models and human post-mortem tissue analysis supporting an RNA editing role in disease manifestation [13,19,44]. Extending our previous research in bulk tissue (cortex) samples from a prion disease model [19], we present cell-type specific data on RNA editing alterations in the context of EAE, as a representative of brain disorder within a degenerative, inflammatory, and autoimmune background.

RNA editing has been proven to play an important role in neuroinflammation by modulating and regulating microglia plasticity and activation [20,21]. Especially in MS, A-I RNA editing in specific *Alu* RNA regions has been reported to be decreased in genes that are known to trigger proinflammatory responses through RIG and TLR3 [12]. In line with these studies, we provide evidence that a loss of RNA editing can lead to more severe disease phenotype, along with increased microglia density, supporting its ‘protective’ and regulatory role in neurodegeneration and neuroinflammation. More specifically, we report, for the first time, high-confidence RNA editing events and microglia editomes during EAE progression, highlighting the cellular processes affected by RNA editing alterations and providing proof of the principle experimental validation of RNA editing manifestations in EAE. Consistent with previous studies [20,44,51,52,53], we report reduced global editing associated with disease progression and identify over-representation of RNA editing events in the 3′UTRs and intronic regions [54]. The observed RNA editing reduction did not correlate with editing-mediating enzyme expression levels, which displayed elevated expression during disease progression (Figure S12). Previous studies have also reported poor or no correlation between RNA editing-mediating enzyme expression levels and editing [46,55], suggesting that the observed reduced editing may be attributed to other factors, resulting in differential access to targeted RNA, due to the pathology.

The 3′UTRs genomic distribution enrichment of editing events in EAE microglia potentially reflects the functional effects of RNA editing on gene expression regulation, as previously suggested [20,56]. Increased exonic editing and higher representation of non-synonymous editing sites, yet at reduced editing frequencies, compared to the naïve condition, may suggest a functional role of these recoding events in disease manifestation. Pathway analysis, performed on murine microglia editomes during EAE progression, confirmed the previous reports on RNA editing contribution in the modulation of inflammation [20] and innate immune responses, including the cytokine and chemokine pathways [29,57]. In addition, previous associations of RNA editing with lysosome function [20] and phagocytosis [56] in microglia and macrophages were highlighted in the context of EAE microglia. Importantly, novel RNA-editing modulated mechanisms affecting microglial functions in EAE progression are suggested through the identification of differentially enriched pathways in pre-clinical and clinical disease stages, compared to the naïve condition, thus paving new ways for additional mechanistic studies in the field. Among these, we highlight the Rap1, AGE-RAGE, and MAPK signaling pathways, which have been associated with Toll-like receptor-triggered inflammatory responses in macrophages/microglia [58,59,60], and the thyroid hormone signaling pathway, which has been shown to modulate microglial migration and phagocytosis [61]. Moreover, we highlight a potential involvement of RNA editing in the modulation of the neurotrophin signaling pathway, affecting microglia–neuron crosstalk [62,63]. Additionally of interest, our data suggests the C-type lectin receptor signaling pathway as an emerging RNA editing modulated contributor in EAE progression, enhancing further studies to extend the currently poor understanding of CLRs involvement in EAE/MS.

We identified the statistically significant differential C-U editing between naïve and acute EAE microglia in the 3′UTR region of the *B2m* transcript, at sites known to undergo C-U editing in mice, suggesting a potential functional role in disease progression [20,56]. *B2m* is a conserved molecule involved in antigen presentation through the cell surface stabilization of major histocompatibility I (MHC-I) and/or MHC-I-like complexes [64]. There are several lines of evidence suggesting a critical role of *B2m* in EAE/MS progression and microglial function. More specifically, *B2m* expression is increased in MOG_35–55_-induced EAE [36] (experimental validation provided in this study) and in cuprizone-induced EAE brain tissue, as well as multiple sclerosis (MS) lesions [65]. Moreover, evidence of direct *B2m* involvement in EAE progression is highlighted by a study where *B2m*-deficient EAE mice show severe disease, increased macrophage infiltration, and enhanced neuronal damage, compared to wild-type animals [48]. On the other hand, there is evidence suggesting that increased *B2m* expression may be linked with the induction of a proinflammatory response that promotes neurodegeneration [66,67]. Our study extends to epitranscriptomic alterations occurring in the *B2m* transcript in EAE microglia, suggesting the functional effects of altered RNA editing in gene expression regulation. We suggest mechanistic insights to the observed increased B2m levels, possibly exerted through miRNA retargeting. Considering that miRNAs impose translational repression or target degradation through binding to corresponding mRNA targets [68] and that editing frequency at the identified position 2: 122,152,740 is increased in EAE microglia, it could be possible that the loss of miR-466b-5p binding, due to *B2m* 3′UTR differential editing, contributes to the increased *B2m* expression in EAE. Moreover, the experimental validation of the statistically significant C-U editing at position 2: 122,152,740 in EAE mice brain tissue may also suggest a similar functional effect exerted not only in microglia, but also in other APOBEC-expressing glial cells, including astrocytes.

Next, we provide an experimental validation of A-I editing in two previously reported positions residing at the *Mpeg1* 3′UTR (19:464262, 19:464309) and further report the differential editing frequencies in EAE microglia for both edited sites. *Mpeg1* encodes perforin 2, a pore-forming cytolytic protein with broad-spectrum activity, which is constitutively expressed in phagocytes and shares high similarity with complement component 9 (C9). Perforin is involved in EAE progression, with a protective immunomodulatory role exerted through regulatory T cells [49]. Interestingly, perforin expression has been shown in microglia from prion-infected mice in regions displaying disease-induced spongiform changes [69], suggesting its involvement in disease-related microglia. The increased expression of *Mpeg1* in EAE microglia further supports this notion [36]. Our data suggest potential regulatory mechanisms of *Mpeg1* expression in EAE microglia. in silico analysis of the unedited and edited *Mpeg1* 3′UTR variants, using the miRDB tool, predicted that editing at position 19:464262 introduces a binding site for mmu-miR-31-5p, while editing at position 19:464309 abolishes the binding site of mmu-miR-6400, present in the unedited variant. Even though miRNA binding is generally associated with repressed target expression, experimental functional assays (e.g. luciferase reporter gene assays) are required to delineate the effects of specific miRNA–mRNA interactions.

Importantly, the identified role of RNA editing in EAE was confirmed by the induction of disease in wild-type and APOBEC-1-deficient mice, which resulted in exaggerated microglia activation accompanied by increased astrogliosis, as well as T cell infiltration and higher clinical scores in EAE APOBEC-1 KO mice, compared to wild-type EAE animals.

Our study provides, for the first time, strong evidence on the involvement of RNA editing in EAE progression, paving the way for additional studies, in order to delineate the underlying RNA-editing modulated disease mechanisms. In spite of its significance, this study has some limitations. In particular, the dataset we utilized for in silico RNA editing analysis comprises pooled sample collections lacking information on potential individual heterogeneity. in silico predictions were experimentally validated for the selected targets using TA cloning and Sanger sequencing entailing the analysis of at least 30 clones per target and sample. The significant finding of this study refers to the observation that APOBEC-1 loss results in more severe EAE. Our study links RNA editing with the microglia functions associated with activation and inflammation in EAE and requires further studies. Considering that RNA editing displays species-specific differences, extrapolation from EAE to MS should be performed with caution, requiring cross-validation of mouse-to-human targets, complemented by comparative transcriptome analyses.

In summary, we established RNA editing involvement in EAE by providing comprehensive microglial editome profiles during EAE progression. The enrichment of the edited transcripts involved in disease-associated processes provides proof-of-principle experimental validation of RDDs in isolated microglia and whole brain tissue samples from wild-type naive and EAE mice. Functional implications of the detected RNA editing differences and the development of more severe diseases in mice deficient in APOBEC-1, the main C-U editing enzyme, supports the ‘protective’ effect of RNA editing in disease progression and severity.

## Data Availability

Available upon request.

## Supplementary Materials

The following supporting information can be downloaded at: https://www.mdpi.com/article/10.3390/cells11223582/s1. Supplementary File S1: Detailed lists of high confidence ADAR (A-I)-mediated RNA editing events identified in naïve, pre-clinical, and acute EAE murine microglia; Supplementary File S2: Detailed lists of high confidence APOBEC (C-U)-mediated RNA editing events identified in naïve, pre-clinical, and acute EAE murine microglia; Supplementary File S3: Supplementary tables and figures in the order mentioned in text: Table S1: Summary of RNA-seq data quality control and alignment data for each sample; Figure S1: Outline of the in-house RNA editing analysis pipeline used in this study; Figure S2: Distribution of the 12 types of RDDs detected in the studied murine microglia datasets. Table S2: Summary of global RNA editing events, mediated by both ADAR and APOBEC editing enzyme families, in naïve and EAE microglia during disease progression and their distribution across functional genomic regions; Table S3: Summary of ADAR-mediated RNA editing events in naïve and EAE microglia during disease progression and their distribution across functional genomic regions; Table S4: Summary of APOBEC-mediated RNA editing events in naïve and EAE microglia during disease progression and their distribution across functional genomic regions; Figure S3: Clustergram depicting the 60 most significant transcripts associated with the top 10 enriched pathways identified for RNA-edited targets in naïve murine microglia; Figure S4: Clustergram depicting the 60 most significant transcripts associated with the top 10 enriched pathways identified for RNA-edited targets in pre-clinical EAE murine microglia; Figure S5: Clustergram depicting the 60 most significant transcripts associated with the top 10 enriched pathways identified for RNA-edited targets in clinical EAE murine microglia; Figure S6: Differential expression of *Mpeg1* and *B2m* transcripts in naïve and acute phase EAE; Table S5: Summary of potentially disease-associated RNA editing events experimentally verified in this study in naïve (Cntrl) and acute phase EAE brain microglia and brain tissue; Figure S7: Detailed Sanger sequencing analysis for detection and quantification of C-U-T RNA editing events in the *B2m* 3′UTR from naïve and acute EAE microglia; Figure S8: Detailed Sanger sequencing analysis for detection and quantification of C-U-T RNA editing events in the *B2m* 3′UTR from naïve and acute EAE brain tissue; Figure S9: Detailed Sanger sequencing analysis for detection and quantification of A-I-G RNA editing events in the *Mpeg1* 3′UTR from naïve and acute EAE microglia; Figure S10: Detailed Sanger sequencing analysis for detection and quantification of A-I-G RNA editing events in the *Mpeg1* 3′UTR from naïve and acute EAE brain tissue; Figure S11: Mean weight variations of APOBEC-1 KO and WT mice during EAE progression; Figure S12: Main RNA-mediating enzymes mRNA expression levels in microglia isolated from naïve, pre-clinical, and clinical EAE mice.

## References

[1] Miedema A., Gerrits E., Brouwer N., Jiang Q., Kracht L., Meijer M., Nutma E., Peferoen-Baert R., Pijnacker A.T.E., Wesseling E.M. (2022). Brain Macrophages Acquire Distinct Transcriptomes in Multiple Sclerosis Lesions and Normal Appearing White Matter. Acta Neuropathol. Commun..

[2] Lassmann H. (2007). Experimental Models of Multiple Sclerosis. Rev. Neurol..

[3] Sawcer S., Franklin R.J.M., Ban M. (2014). Multiple Sclerosis Genetics. Lancet Neurol..

[4] Santiago J.A., Bottero V., Potashkin J.A. (2017). Biological and Clinical Implications of Comorbidities in Parkinson’s Disease. Front. Aging Neurosci..

[5] Karagianni K., Pettas S., Christoforidou G., Kanata E., Bekas N., Xanthopoulos K., Dafou D., Sklaviadis T. (2022). A Systematic Review of Common and Brain-Disease-Specific RNA Editing Alterations Providing Novel Insights into Neurological and Neurodegenerative Disease Manifestations. Biomolecules.

[6] Farajollahi S., Maas S. (2010). Molecular Diversity through RNA Editing: A Balancing Act. Trends Genet..

[7] Nishikura K. (2015). A-to-I Editing of Coding and Non-Coding RNAs by ADARs. Nat. Rev. Mol. Cell Biol..

[8] Licht K., Jantsch M.F. (2016). Rapid and Dynamic Transcriptome Regulation by RNA Editing and RNA Modifications. J. Cell Biol..

[9] Tan M.H., Li Q., Shanmugam R., Piskol R., Kohler J., Young A.N., Liu K.I., Zhang R., Ramaswami G., Ariyoshi K. (2017). Dynamic Landscape and Regulation of RNA Editing in Mammals. Nature.

[10] Salter J.D., Bennett R.P., Smith H.C. (2016). The APOBEC Protein Family: United by Structure, Divergent in Function. Trends Biochem. Sci..

[11] Picardi E., Manzari C., Mastropasqua F., Aiello I., D’Erchia A.M., Pesole G. (2015). Profiling RNA Editing in Human Tissues: Towards the Inosinome Atlas. Sci. Rep..

[12] Tossberg J.T., Heinrich R.M., Farley V.M., Crooke P.S., Aune T.M. (2020). Adenosine-to-Inosine RNA Editing of Alu Double-Stranded (Ds)RNAs Is Markedly Decreased in Multiple Sclerosis and Unedited Alu DsRNAs Are Potent Activators of Proinflammatory Transcriptional Responses. J. Immunol..

[13] Srivastava P.K., Bagnati M., Delahaye-Duriez A., Ko J.H., Rotival M., Langley S.R., Shkura K., Mazzuferi M., Danis B., Van Eyll J. (2017). Genome-Wide Analysis of Differential RNA Editing in Epilepsy. Genome Res..

[14] Tran S.S., Jun H.I., Bahn J.H., Azghadi A., Ramaswami G., Van Nostrand E.L., Nguyen T.B., Hsiao Y.H.E., Lee C., Pratt G.A. (2018). Widespread RNA Editing Dysregulation in Brains from Autistic Individuals. Nat. Neurosci..

[15] Flomen R., Makoff A. (2011). Increased RNA Editing in EAAT2 Pre-MRNA from Amyotrophic Lateral Sclerosis Patients: Involvement of a Cryptic Polyadenylation Site. Neurosci. Lett..

[16] Akbarian S., Smith M.A., Jones E.G. (1995). Editing for an AMPA Receptor Subunit RNA in Prefrontal Cortex and Striatum in Alzheimer’s Disease, Huntington’s Disease and Schizophrenia. Brain Res..

[17] Gaisler-Salomon I., Kravitz E., Feiler Y., Safran M., Biegon A., Amariglio N., Rechavi G. (2014). Hippocampus-Specific Deficiency in RNA Editing of GluA2 in Alzheimer’s Disease. Neurobiol. Aging.

[18] Khermesh K., D’Erchia A.M., Barak M., Annese A., Wachtel C., Levanon E.Y., Picardi E., Eisenberg E. (2016). Reduced Levels of Protein Recoding by A-to-I RNA Editing in Alzheimer’s Disease. RNA.

[19] Kanata E., Llorens F., Dafou D., Dimitriadis A., Thüne K., Xanthopoulos K., Bekas N., Espinosa J.C., Schmitz M., Marín-Moreno A. (2019). RNA Editing Alterations Define Manifestation of Prion Diseases. Proc. Natl. Acad. Sci. USA.

[20] Cole D.C., Chung Y., Gagnidze K., Hajdarovic K.H., Rayon-Estrada V., Harjanto D., Bigio B., Gal-Toth J., Milner T.A., McEwen B.S. (2017). Loss of APOBEC1 RNA-Editing Function in Microglia Exacerbates Age-Related CNS Pathophysiology. Proc. Natl. Acad. Sci. USA.

[21] Guo X., Wiley C.A., Steinman R.A., Sheng Y., Ji B., Wang J., Zhang L., Wang T., Zenatai M., Billiar T.R. (2021). Aicardi-Goutières Syndrome-Associated Mutation at ADAR1 Gene Locus Activates Innate Immune Response in Mouse Brain. J. Neuroinflammation.

[22] Rice G.I., Kasher P.R., Forte G.M.A., Mannion N.M., Greenwood S.M., Szynkiewicz M., Dickerson J.E., Bhaskar S.S., Zampini M., Briggs T.A. (2012). Mutations in ADAR1 Cause Aicardi-Goutières Syndrome Associated with a Type I Interferon Signature. Nat. Genet..

[23] Mannion N.M., Greenwood S.M., Young R., Cox S., Brindle J., Read D., Nellåker C., Vesely C., Ponting C.P., McLaughlin P.J. (2014). The RNA-Editing Enzyme ADAR1 Controls Innate Immune Responses to RNA. Cell Rep..

[24] Liddicoat B.J., Piskol R., Chalk A.M., Ramaswami G., Higuchi M., Hartner J.C., Li J.B., Seeburg P.H., Walkley C.R. (2015). RNA Editing by ADAR1 Prevents MDA5 Sensing of Endogenous DsRNA as Nonself. Science.

[25] Pestal K., Funk C.C., Snyder J.M., Price N.D., Treuting P.M., Stetson D.B. (2015). Isoforms of RNA-Editing Enzyme ADAR1 Independently Control Nucleic Acid Sensor MDA5-Driven Autoimmunity and Multi-Organ Development. Immunity.

[26] Eisenberg E., Levanon E.Y. (2018). A-to-I RNA Editing—Immune Protector and Transcriptome Diversifier. Nat. Rev. Genet..

[27] Samuel C.E. (2019). Adenosine Deaminase Acting on RNA (ADAR1), a Suppressor of Double-Stranded RNA–Triggered Innate Immune Responses. J. Biol. Chem..

[28] Li Q., Gloudemans M.J., Geisinger J.M., Fan B., Aguet F., Sun T., Ramaswami G., Li Y.I., Ma J.-B., Pritchard J.K. (2022). RNA Editing Underlies Genetic Risk of Common Inflammatory Diseases. Nature.

[29] Alqassim E.Y., Sharma S., Khan A.N.M.N.H., Emmons T.R., Cortes Gomez E., Alahmari A., Singel K.L., Mark J., Davidson B.A., Robert McGray A.J. (2021). RNA Editing Enzyme APOBEC3A Promotes Pro-Inflammatory M1 Macrophage Polarization. Commun. Biol..

[30] Xiang R., Liu Y., Fan L., Jiang B., Wang F. (2022). RNA Adenosine Deaminase (ADAR1) Alleviates High-Fat Diet-Induced Nonalcoholic Fatty Liver Disease by Inhibiting NLRP3 Inflammasome. Lab. Investig..

[31] Pettas S., Karagianni K., Kanata E., Chatziefstathiou A., Christoudia N., Xanthopoulos K., Sklaviadis T., Dafou D. (2022). Profiling Microglia through Single-Cell RNA Sequencing over the Course of Development, Aging, and Disease. Cells.

[32] Jordão M.J.C., Sankowski R., Brendecke S.M., Locatelli G., Tai Y.H., Tay T.L., Schramm E., Armbruster S., Hagemeyer N., Sagar (2019). Single-Cell Profiling Identifies Myeloid Cell Subsets with Distinct Fates during Neuroinflammation. Science.

[33] Voet S., Prinz M., van Loo G. (2019). Microglia in Central Nervous System Inflammation and Multiple Sclerosis Pathology. Trends Mol. Med..

[34] Li Q., Barres B.A. (2017). Microglia and Macrophages in Brain Homeostasis and Disease. Nat. Rev. Immunol..

[35] Luo C., Jian C., Liao Y., Huang Q., Wu Y., Liu X., Zou D., Wu Y. (2017). The Role of Microglia in Multiple Sclerosis. Neuropsychiatr. Dis. Treat..

[36] Lewis N.D., Hill J.D., Juchem K.W., Stefanopoulos D.E., Modis L.K. (2014). RNA Sequencing of Microglia and Monocyte-Derived Macrophages from Mice with Experimental Autoimmune Encephalomyelitis Illustrates a Changing Phenotype with Disease Course. J. Neuroimmunol..

[37] Chen E.Y., Tan C.M., Kou Y., Duan Q., Wang Z., Meirelles G.V., Clark N.R., Ma’ayan A. (2013). Enrichr: Interactive and Collaborative HTML5 Gene List Enrichment Analysis Tool. BMC Bioinform..

[38] Kuleshov M.V., Jones M.R., Rouillard A.D., Fernandez N.F., Duan Q., Wang Z., Koplev S., Jenkins S.L., Jagodnik K.M., Lachmann A. (2016). Enrichr: A Comprehensive Gene Set Enrichment Analysis Web Server 2016 Update. Nucleic Acids Res..

[39] Hirano K.I., Young S.G., Farese R.V., Ng J., Sande E., Warburton C., Powell-Braxton L.M., Davidson N.O. (1996). Targeted Disruption of the Mouse Apobec-1 Gene Abolishes Apolipoprotein B MRNA Editing and Eliminates Apolipoprotein B48. J. Biol. Chem..

[40] Grigoriadis N., Lourbopoulos A., Lagoudaki R., Frischer J.M., Polyzoidou E., Touloumi O., Simeonidou C., Deretzi G., Kountouras J., Spandou E. (2011). Variable Behavior and Complications of Autologous Bone Marrow Mesenchymal Stem Cells Transplanted in Experimental Autoimmune Encephalomyelitis. Exp. Neurol..

[41] Theotokis P., Kleopa K.A., Touloumi O., Lagoudaki R., Lourbopoulos A., Nousiopoulou E., Kesidou E., Poulatsidou K.N., Dardiotis E., Hadjigeorgiou G. (2015). Connexin43 and Connexin47 Alterations after Neural Precursor Cells Transplantation in Experimental Autoimmune Encephalomyelitis. Glia.

[42] Theotokis P., Touloumi O., Lagoudaki R., Nousiopoulou E., Kesidou E., Siafis S., Tselios T., Lourbopoulos A., Karacostas D., Grigoriadis N. (2016). Nogo Receptor Complex Expression Dynamics in the Inflammatory Foci of Central Nervous System Experimental Autoimmune Demyelination. J. Neuroinflammation.

[43] Gardner O.K., Wang L., van Booven D., Whitehead P.L., Hamilton-Nelson K.L., Adams L.D., Starks T.D., Hofmann N.K., Vance J.M., Cuccaro M.L. (2019). RNA Editing Alterations in a Multi-Ethnic Alzheimer Disease Cohort Converge on Immune and Endocytic Molecular Pathways. Hum. Mol. Genet..

[44] Dick A.L.W., Khermesh K., Paul E., Stamp F., Levanon E.Y., Chen A. (2019). Adenosine-to-Inosine RNA Editing within Corticolimbic Brain Regions Is Regulated in Response to Chronic Social Defeat Stress in Mice. Front. Psychiatry.

[45] Roth S.H., Danan-Gotthold M., Ben-Izhak M., Rechavi G., Cohen C.J., Louzoun Y., Levanon E.Y. (2018). Increased RNA Editing May Provide a Source for Autoantigens in Systemic Lupus Erythematosus. Cell Rep..

[46] Moore S., Alsop E., Lorenzini I., Starr A., Rabichow B.E., Mendez E., Levy J.L., Burciu C., Reiman R., Chew J. (2019). ADAR2 Mislocalization and Widespread RNA Editing Aberrations in C9orf72-Mediated ALS/FTD. Acta Neuropathol..

[47] Vlachogiannis N.I., Gatsiou A., Silvestris D.A., Stamatelopoulos K., Tektonidou M.G., Gallo A., Sfikakis P.P., Stellos K. (2020). Increased Adenosine-to-Inosine RNA Editing in Rheumatoid Arthritis. J. Autoimmun..

[48] Linker R.A., Reinhardt M., Bendszus M., Ladewig G., Briel A., Schirner M., Mäurer M., Hauff P. (2005). In Vivo Molecular Imaging of Adhesion Molecules in Experimental Autoimmune Encephalomyelitis (EAE). J. Autoimmun..

[49] Beeston T., Smith T.R.F., Maricic I., Tang X., Kumar V. (2010). Involvement of IFN-γ and Perforin, but Not Fas/FasL Interactions in Regulatory T Cell-Mediated Suppression of Experimental Autoimmune Encephalomyelitis. J. Neuroimmunol..

[50] Chalk A.M., Taylor S., Heraud-Farlow J.E., Walkley C.R. (2019). The Majority of A-to-I RNA Editing Is Not Required for Mammalian Homeostasis. Genome Biol..

[51] Danecek P., Nellåker C., McIntyre R.E., Buendia-Buendia J.E., Bumpstead S., Ponting C.P., Flint J., Durbin R., Keane T.M., Adams D.J. (2012). High Levels of RNA-Editing Site Conservation amongst 15 Laboratory Mouse Strains. Genome Biol..

[52] Harjanto D., Papamarkou T., Oates C.J., Rayon-Estrada V., Papavasiliou F.N., Papavasiliou A. (2016). RNA Editing Generates Cellular Subsets with Diverse Sequence within Populations. Nat. Commun..

[53] Picardi E., Horner D.S., Pesole G. (2017). Single-Cell Transcriptomics Reveals Specific RNA Editing Signatures in the Human Brain. RNA.

[54] Hwang T., Park C.K., Leung A.K.L., Gao Y., Hyde T.M., Kleinman J.E., Rajpurohit A., Tao R., Shin J.H., Weinberger D.R. (2016). Dynamic Regulation of RNA Editing in Human Brain Development and Disease. Nat. Neurosci..

[55] Quinones-Valdez G., Tran S.S., Jun H.I., Bahn J.H., Yang E.W., Zhan L., Brümmer A., Wei X., Van Nostrand E.L., Pratt G.A. (2019). Regulation of RNA Editing by RNA-Binding Proteins in Human Cells. Commun. Biol..

[56] Rayon-Estrada V., Harjanto D., Hamilton C.E., Berchiche Y.A., Gantman E.C., Sakmar T.P., Bulloch K., Gagnidze K., Harroch S., McEwen B.S. (2017). Epitranscriptomic Profiling across Cell Types Reveals Associations between APOBEC1-Mediated RNA Editing, Gene Expression Outcomes, and Cellular Function. Proc. Natl. Acad. Sci. USA.

[57] Pujantell M., Riveira-Muñoz E., Badia R., Castellví M., Garcia-Vidal E., Sirera G., Puig T., Ramirez C., Clotet B., Esté J.A. (2017). RNA Editing by ADAR1 Regulates Innate and Antiviral Immune Functions in Primary Macrophages. Sci. Rep..

[58] Tang S., Chen T., Yu Z., Zhu X., Yang M., Xie B., Li N., Cao X., Wang J. (2014). RasGRP3 Limits Toll-like Receptor-Triggered Inflammatory Response in Macrophages by Activating Rap1 Small GTPase. Nat. Commun..

[59] Shen C., Ma Y., Zeng Z., Yin Q., Hong Y., Hou X., Liu X. (2017). RAGE-Specific Inhibitor FPS-ZM1 Attenuates AGEs-Induced Neuroinflammation and Oxidative Stress in Rat Primary Microglia. Neurochem. Res..

[60] Bachstetter A.D., Xing B., de Almeida L., Dimayuga E.R., Watterson D.M., Van Eldik L.J. (2011). Microglial P38α MAPK Is a Key Regulator of Proinflammatory Cytokine Up-Regulation Induced by Toll-like Receptor (TLR) Ligands or Beta-Amyloid (Aβ). J. Neuroinflamm..

[61] Mori Y., Tomonaga D., Kalashnikova A., Furuya F., Akimoto N., Ifuku M., Okuno Y., Beppu K., Fujita K., Katafuchi T. (2015). Effects of 3,3′,5-Triiodothyronine on Microglial Functions. Glia.

[62] Prowse N., Hayley S. (2021). Microglia and BDNF at the Crossroads of Stressor Related Disorders: Towards a Unique Trophic Phenotype. Neurosci. Biobehav. Rev..

[63] Pöyhönen S., Er S., Domanskyi A., Airavaara M. (2019). Effects of Neurotrophic Factors in Glial Cells in the Central Nervous System: Expression and Properties in Neurodegeneration and Injury. Front. Physiol..

[64] Li H., Chen C., Yao H., Li X., Yang N., Qiao J., Xu K., Zeng L. (2016). Identification of Suitable Reference Genes for MRNA Studies in Bone Marrow in a Mouse Model of Hematopoietic Stem Cell Transplantation. Transplant. Proc..

[65] Martin N.A., Nawrocki A., Molnar V., Elkjaer M.L., Thygesen E.K., Palkovits M., Acs P., Sejbaek T., Nielsen H.H., Hegedus Z. (2018). Orthologous Proteins of Experimental De- and Remyelination Are Differentially Regulated in the CSF Proteome of Multiple Sclerosis Subtypes. PLoS ONE.

[66] Starkey H.D.V., Van Kirk C.A., Bixler G.V., Imperio C.G., Kale V.P., Serfass J.M., Farley J.A., Yan H., Warrington J.P., Han S. (2012). Neuroglial Expression of the Mhci Pathway and Pirb Receptor Is Upregulated in the Hippocampus with Advanced Aging. J. Mol. Neurosci..

[67] Yousef H., Conboy M.J., Morgenthaler A., Schlesinger C., Bugaj L., Paliwal P., Greer C., Conboy I.M., Schaffer D. (2015). Systemic Attenuation of the TGF-β Pathway by a Single Drug Simultaneously Rejuvenates Hippocampal Neurogenesis and Myogenesis in the Same Old Mammal. Oncotarget.

[68] Hammond T.R.R., McEllin B., Morton P.D.D., Raymond M., Dupree J., Gallo V. (2015). Endothelin-B Receptor Activation in Astrocytes Regulates the Rate of Oligodendrocyte Regeneration during Remyelination. Cell Rep..

[69] Kopacek J., Sakaguchi S., Shigematsu K., Nishida N., Atarashi R., Nakaoke R., Moriuchi R., Niwa M., Katamine S. (2000). Upregulation of the Genes Encoding Lysosomal Hydrolases, a Perforin-Like Protein, and Peroxidases in the Brains of Mice Affected with an Experimental Prion Disease. J. Virol..

