# Supplementary material for: RNA Editing Alterations Define Disease Manifestations in the Progression of Experimental Autoimmune Encephalomyelitis (EAE)
Source: Cells. 2022 Nov 12;11(22):3582. doi: 10.3390/cells11223582 (PMC9688714; doi:10.3390/cells11223582)
Supplement: Supplementary file 1 [file cells-11-03582-s001.zip › Supplementary File S3.pdf]

Table S1. Summary of RNA-seq data quality control and alignment data for each sample used in this study.

Condition	Sample	Starting Reads	Reads after Quality Control	Overall Read Mapping Rate
**Naive**	Mi0	19306727	18438313	95.8% (72.0% concordant)
**Pre-clinical**	Mi8	19053077	18186087	95.7% (72.4% concordant)
**Clinical**	Mi14	20952061	20013343	95.9% (71.3% concordant)

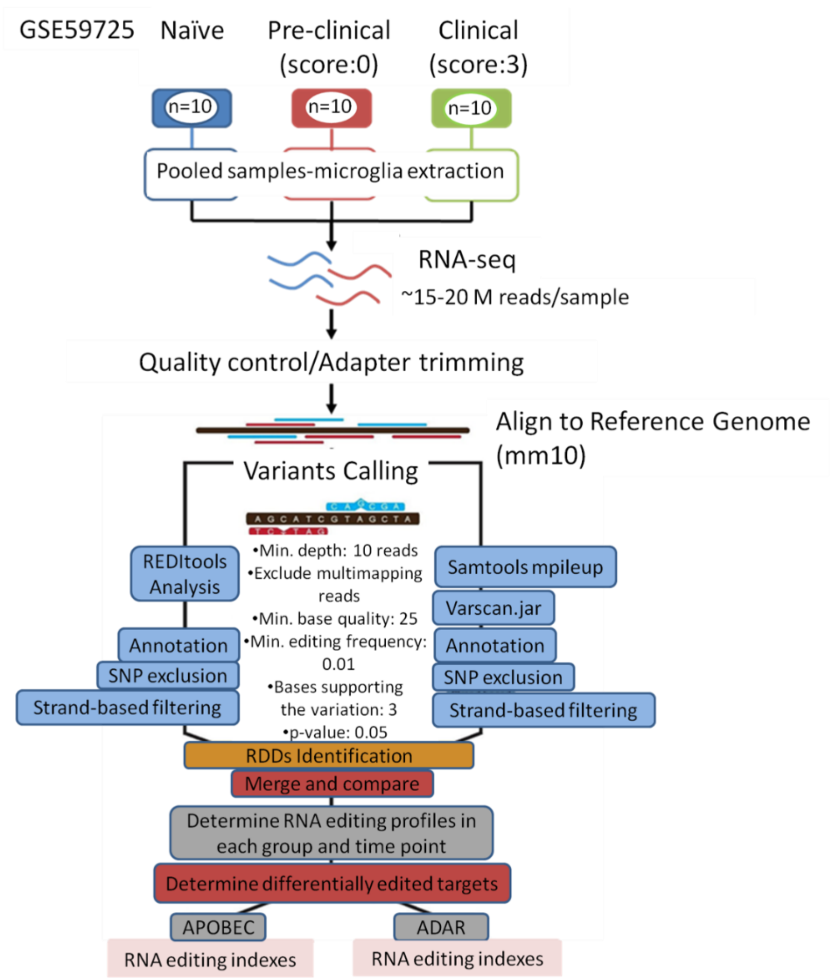

Figure S1. Outline of the in-house RNA editing analysis pipeline used in this study.

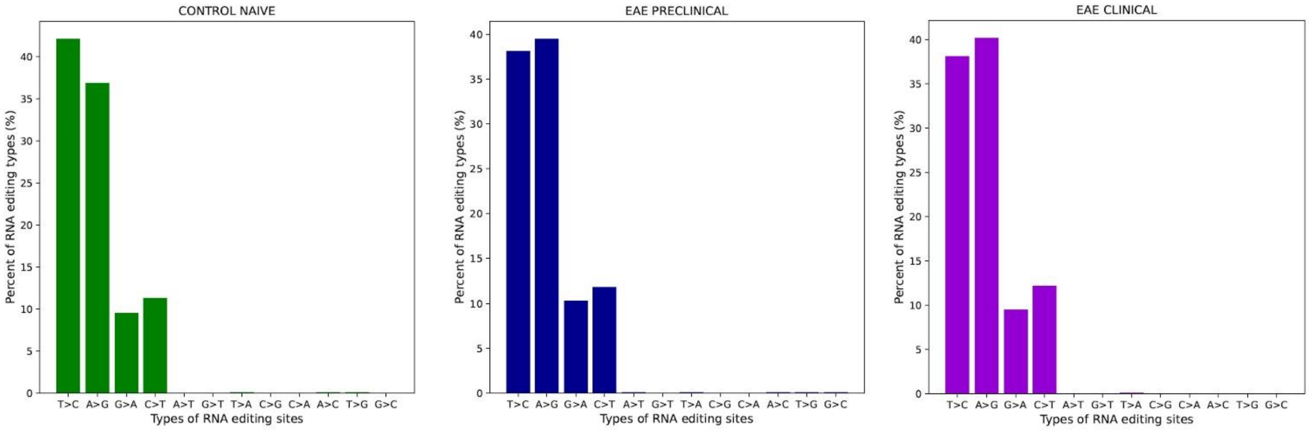

Figure S2: Distribution of the 12 types of RDDs detected in the studied murine microglia dataset following SPRINT analysis. Data represent the percentage of inferred RDDs detected in naive, EAE pre-clinical and EAE clinical disease stage microglia.

Table S2. Summary of global RNA editing events, mediated by both ADAR and APOBEC editing enzyme families, in naïve and EAE microglia during disease progression and their distribution across functional genomic regions. Numbers outside parentheses present absolute numbers of RNA editing events; corresponding percent (%) representations relative to total editing events identified per condition are shown within parentheses.

**Condition**	**Intergenic**	**Other (upstream/ downstream)**	**3'UTR**	**Exonic**	**Intronic**	**ncRNA-exonic**	**ncRNA-intronic**	**5'UTR**	**Total**
**Naïve**	0 (0.0%)	52 (4.2%)	309 (25.2%)	189 (15.4%)	569 (46.4%)	55 (4.5%)	31 (2.5%)	20 (1.6%)	**1225 (100%)**
**Pre-clinial**	161 (14.2%)	31 (2.7%)	281 (24.8%)	160 (14.1%)	408 (36.0%)	53 (4.7%)	27 (2.4%)	13 (1.1%)	**1134 (100%)**
**Clinical (Acute)**	42 (4.7%)	33 (3.7%)	334 (37.5%)	151 (16.9%)	265 (29.7%)	25 (2.8%)	25 (2.8%)	16 (1.8%)	**891 (100%)**

Table S3. Summary of ADAR-mediated RNA editing events in naïve and EAE microglia during disease progression and their distribution across functional genomic regions. Numbers outside parentheses present absolute numbers of RNA editing events; corresponding percent (%) representations relative to total A-I editing events identified per condition are shown within parentheses.

**Condition**	**Intergenic**	**Other (upstream/ downstream)**	**3'UTR**	**Exonic**	**Intronic**	**ncRNA-exonic**	**ncRNA-intronic**	**5'UTR**	**Total**
**Naïve**	0 (0.0%)	36 (4.6%)	180 (23.2%)	119 (15.4%)	388 (50.1%)	27 (3.5%)	20 (2.6%)	5 (0.6%)	**775 (100%)**
**Pre-clinial**	105 (14.1%)	20 (2.7%)	174 (23.4%)	111 (14.9%)	278 (37.4%)	33 (4.4%)	16 (2.2%)	6 (0.8%)	**743 (100%)**
**Clinical (Acute)**	0 (0.0%)	23 (4.2%)	217 (39.7%)	111 (20.3%)	165 (30.2%)	13 (2.4%)	12 (2.2%)	5 (0.9%)	**546 (100%)**

Table S4. Summary of APOBEC-mediated RNA editing events in naïve and EAE microglia during disease progression and their distribution across functional genomic regions. Numbers outside parentheses present absolute numbers of RNA editing events; corresponding percent (%) representations relative to total C-U editing events identified per condition are shown within parentheses.

**Condition**	**Intergenic**	**Other (upstream/ downstream)**	**3'UTR**	**Exonic**	**Intronic**	**ncRNA-exonic**	**ncRNA-intronic**	**5'UTR**	**Total**
**Naïve**	0 (0.0%)	16 (3.6%)	129 (28.7%)	70 (15,6%)	181 (40,2%)	28 (6,2%)	11 (2,4%)	15 (3,3%)	**450 (100%)**
**Pre-clinial**	56 (14.3%)	11 (2.8%)	107 (27.4%)	49 (12,5%)	130 (33,2%)	20 (5,1%)	11 (2,8%)	7 (1,8%)	**391 (100%)**
**Clinical (Acute)**	42 (12.2%)	10 (2.9%)	117 (33.9%)	40 (11,6%)	100 (29%)	12 (3,5%)	13 (3,8%)	11 (3,2%)	**345 (100%)**

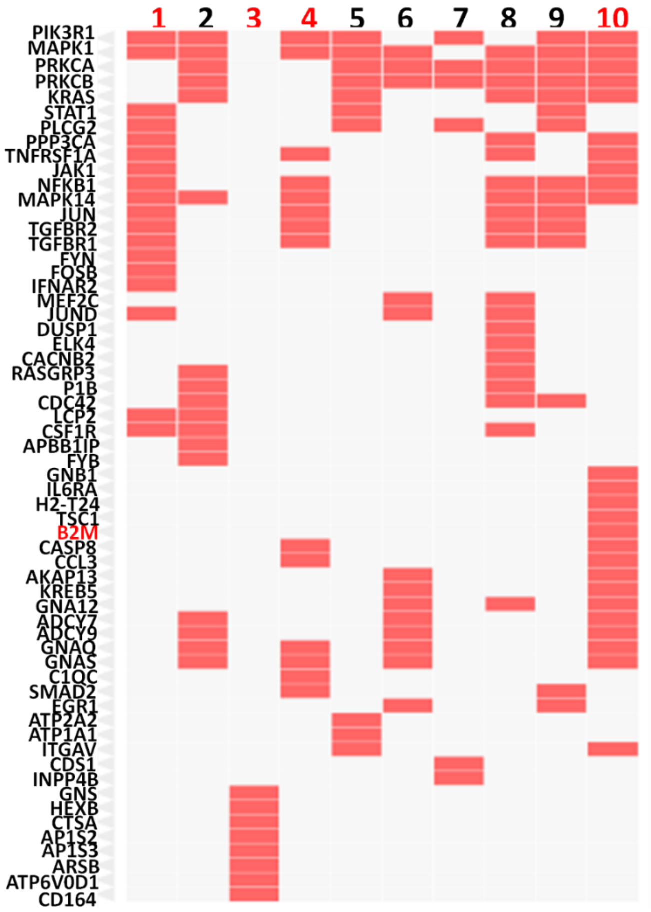

Figure S3. Cluster gram depicting the 60 most significant transcripts associated with the top-10 enriched pathways identified for RNA-edited targets in naïve murine microglia. Pathways are depicted in columns (1: Osteoclast differentiation, p-value: 9.2×10^{-9}, 2: Rap1 signaling, p-value: 1.1×10^{-7} 3: Lysosome, p-value: 1.3×10^{-7}, 4: Chagas disease, p-value: 1.6×10^{-7}, 5: Thyroid hormone signaling, p-value: 8.6×10^{-7}, 6: Parathyroid hormone synthesis, secretion and action, p-value: 6.3×10^{-6}, 7: Phosphatidylinositol signaling system, p-value: 9.2×10^{-6}, 8: MAPK signaling, p-value:1×10^{-5}, 9: AGE-RAGE signaling pathway in diabetic complications, p-value: 1.3×10^{-5}, 10: Human cytomegalovirus infection, p-value: 1.6×10^{-5}). Pathways also identified as enriched at pre-clinical or clinical EAE stages are shown in red. Transcripts are shown in rows. Colored cells indicate the involvement of the specific transcript in the corresponding pathway. The B2m transcript, selected in this study for experimental validation of RNA editing alterations in EAE, is shown in red.

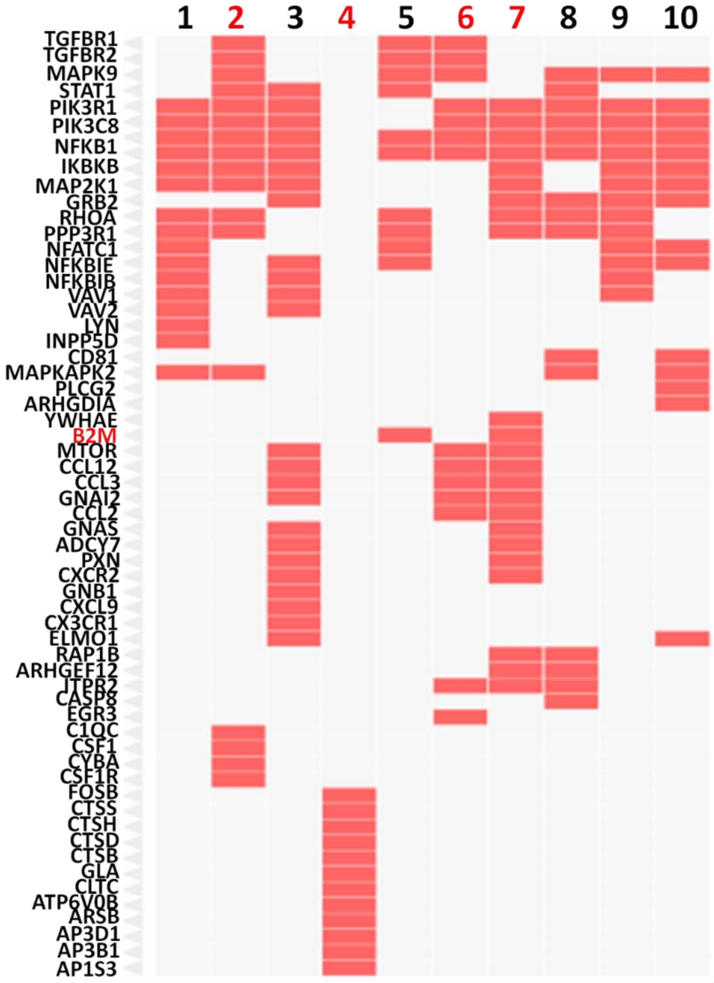

Figure S4. Cluster gram depicting the 60 most significant transcripts associated with the top-10 enriched pathways identified for RNA-edited targets in pre-clinical EAE murine microglia. Pathways are depicted in columns (1: B cell receptor signaling, p-value: 1.9x10-10, 2: Osteoclast differentiation, p-value: 3.1x10-10, 3: Chemokine signaling, p-value: 2.97x10-9, 4: Lysosome, p-value: 6.2x10-9, 5: Th17 cell differentiation, p-value: 5.2x10-8, 6: Chagas disease, p-value: 3.5x10-8, 7: Human cytomegalovirus infection, p-value: 5.8x10-7, 8: C-type lectin receptor signaling, p-value: 1.1x10-6, 9: T cell receptor signaling, p-value: 1.5x10-6, 10: Neurotrophin signaling, p-value: 3.2x10-6). Pathways also identified as enriched at naïve murine microglia or at the clinical EAE stage are shown in red. Transcripts are shown in rows. Colored cells indicate the involvement of the specific transcript in the corresponding pathway. The B2m transcript, selected in this study for experimental validation of RNA editing alterations in EAE, is shown in red.

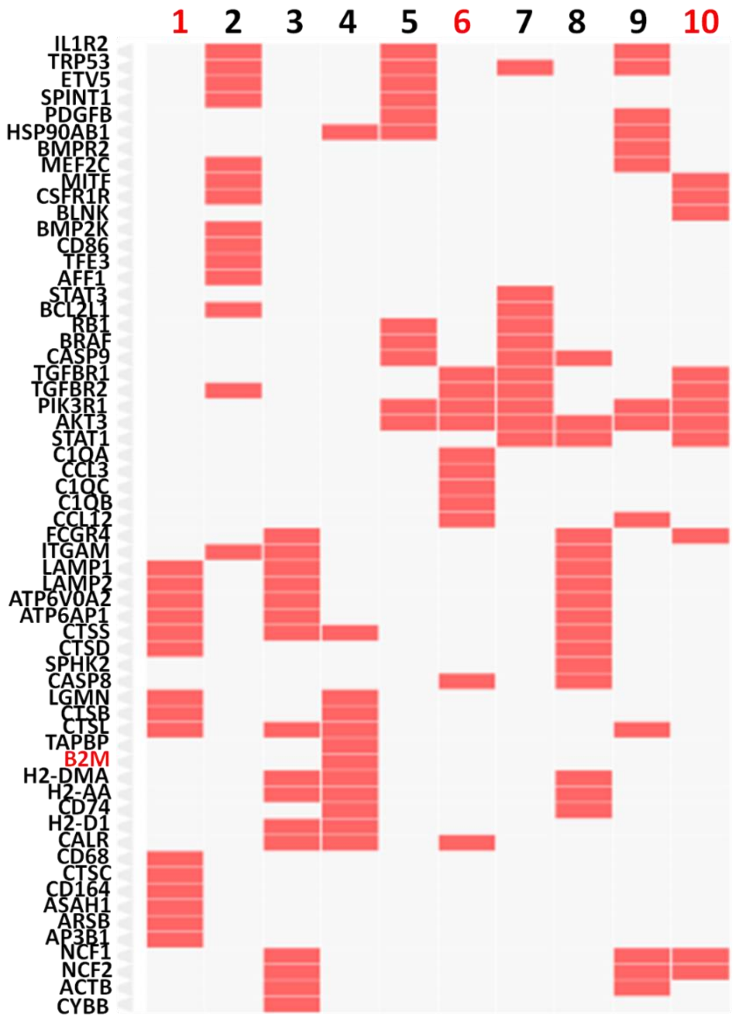

Figure S5. Cluster gram depicting the 60 most significant transcripts associated with the top-10 enriched pathways identified for RNA-edited targets in clinical EAE murine microglia. Pathways are depicted in columns (1: Lysosome, p-value: 1.4x10-17, 2: Transcriptional misregulation in cancer, p-value: 7.7x10-8, 3: Phagosome, p-value: 2.7x10-7, 4: Antigen processing and presentation, p-value: 1.8x10-6,5: Prostate cancer, p-value: 4.2x10-6, 6: Chagas disease, p-value: 8.2x10-6, 7: Pancreatic cancer, p-value: 9.5x10-6, 8: Tuberculosis, p-value: 1.6x10-5, 9: Fluid shear stress and atherosclerosis, p-value: 1.7x10-5, 10: Osteoclast differentiation, p-value: 1.98x10-5). Pathways also identified as enriched at naïve murine microglia or at pre- clinical EAE stage are shown in red. Transcripts are shown in rows. Colored cells indicate the involvement of the specific transcript in the corresponding pathway. The B2m transcript, selected in this study for experimental validation of RNA editing alterations in EAE, is shown in red.

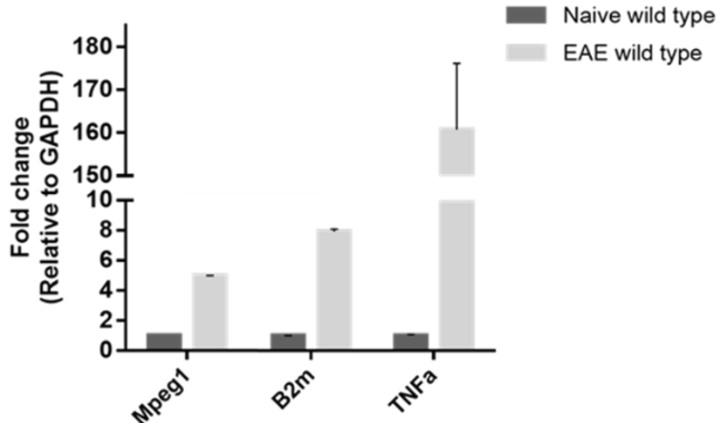

Figure S6. The Mpeg1 and B2m transcripts are differentially expressed in naïve and acute phase EAE. Quantitative real-time PCR validation of transcriptome data for selected edited genes between naïve and acute phase of EAE induced in C57BL/6 wild type female mice. Relative expression was normalized to GAPDH housekeeping gene. TNFa was used to confirm inflammation in EAE. Data are presented as mean±sdev from technical replicates.

Table S5. Summary of potentially disease-associated RNA editing events experimentally verified in this study, in naïve (Cntrl) and acute phase EAE brain microglia and brain tissue. #: Coordinates given relative to murine mm10 Reference Genome, $: Data are presented as mean percentage of editing frequency ± Standard Error, &: For Statistical analyses, one-tailed unpaired t-test was performed. Significance symbols are as follows: * $p \leq 0.05$, ** $p \leq 0.01$, *** $p \leq 0.001$. Exact p values determined in each case are given within parentheses. a: Presented data refer to four technical replicates of brain microglia samples per condition (EAE, Cntr), including the analysis of at least 20 (EAE) or 8 (Cntr) clones per replicate, b: Presented data refer to three biological replicates of brain tissue samples per condition (n=3 EAE, n= 3 Cntr), including the analysis of at least 30 clones per sample, c: Presented data refer to three technical replicates of brain microglia samples per condition (EAE, Cntr); editing frequencies were calculated from corresponding Sanger sequencing chromatograms of PCR amplified cDNAs encompassing part of the target 3'UTR flanking the predicted editing sites. ImageJ was used to calculate editing frequencies as the percentage of G peaks relative to A peaks per editing site. d: Representative chromatograms and clone alignments of these experimental validations are provided in Supplementary Figures S7-8. e: Representative chromatograms and clone alignments of these experimental validations are provided in Supplementary Figures S9-10.

**Condition**	**Target**	**Coordinate[#]**	**Editing Type/ Enzyme Family**	**Editing Detection & Quantification[$, &]**	
				**Brain Microglia**	**Brain Tissue**
**Cntr**	*B2m*	2:122152682	C-U-T/ APOBEC	0.0 ± 0.0[a,d]	0.0 ± 0.0[b,d]
**EAE**	*B2m*	2:122152682	C-U-T/APOBEC	3.4 ± 1.2[a,d] * (p=0.0133)	0.8 ± 0.8[b,d] ns (p=0.1870)
**Cntr**	*B2m*	2:122152740	C-U-T/APOBEC	5.9 ± 3.4[a,d]	0.0 ± 0.0[b,d]
**EAE**	*B2m*	2:122152740	C-U-T/APOBEC	16.5 ± 3.6[a,d] * (p=0.0388)	16.8 ± 7.2[b,d] * (p=0.0403)
**Cntr**	*B2m*	2: 122152871	C-U-T/APOBEC	5.9 ± 3.4[a,d]	0.0 ± 0.0[b,d]
**EAE**	*B2m*	2: 122152871	C-U-T/APOBEC	3.5 ± 1.6 ns (p=0.2723)	3.4 ± 0.79[b,d] ** (p=0.0063)
**Cntr**	*Mpeg1*	19:464262	A-I-G/ADAR	20.3 ± 3.5[c,e]	21.5 ± 3.1[b,e]
**EAE**	*Mpeg1*	19:464262	A-I-G/ADAR	43.3 ± 3.3[c,e] ** (p=0.0044)	39.4 ± 4.0[b,e] * (p=0.0122)
**Cntr**	*Mpeg1*	19:464309	A-I-G/ADAR	6.7 ± 1.7[c,e]	13.8 ± 3.4[b,e]
**EAE**	*Mpeg1*	19:464309	A-I-G/ADAR	21.3 ± 1.9[c,e] ** (p=0.0021)	22.6 ± 1.7[b,e] * (p=0.0385)

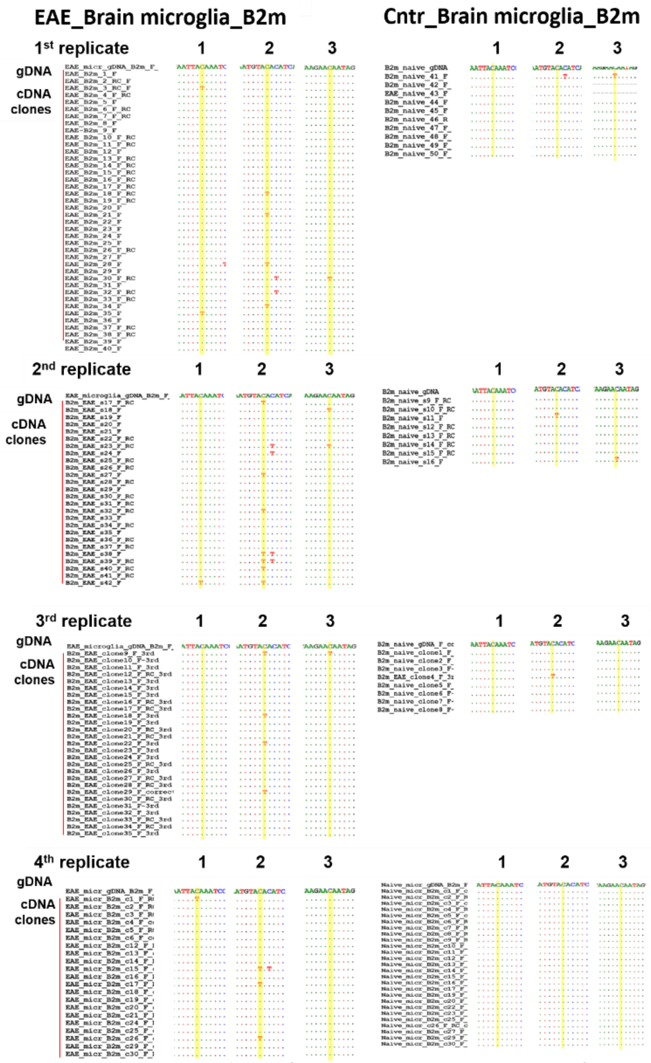

Figure S7. Detailed Sanger sequencing analysis for detection and quantification of C-U-T RNA editing events in the B2m 3'UTR from naïve and acute EAE microglia (4 technical replicates). Alignments of cDNA clones relative to the corresponding reference gDNA sequence. Positions undergoing C-U-T editing as shown in yellow highlight. 1: 2:122152682, 2: 2:122152740, 3: 2:122152871.

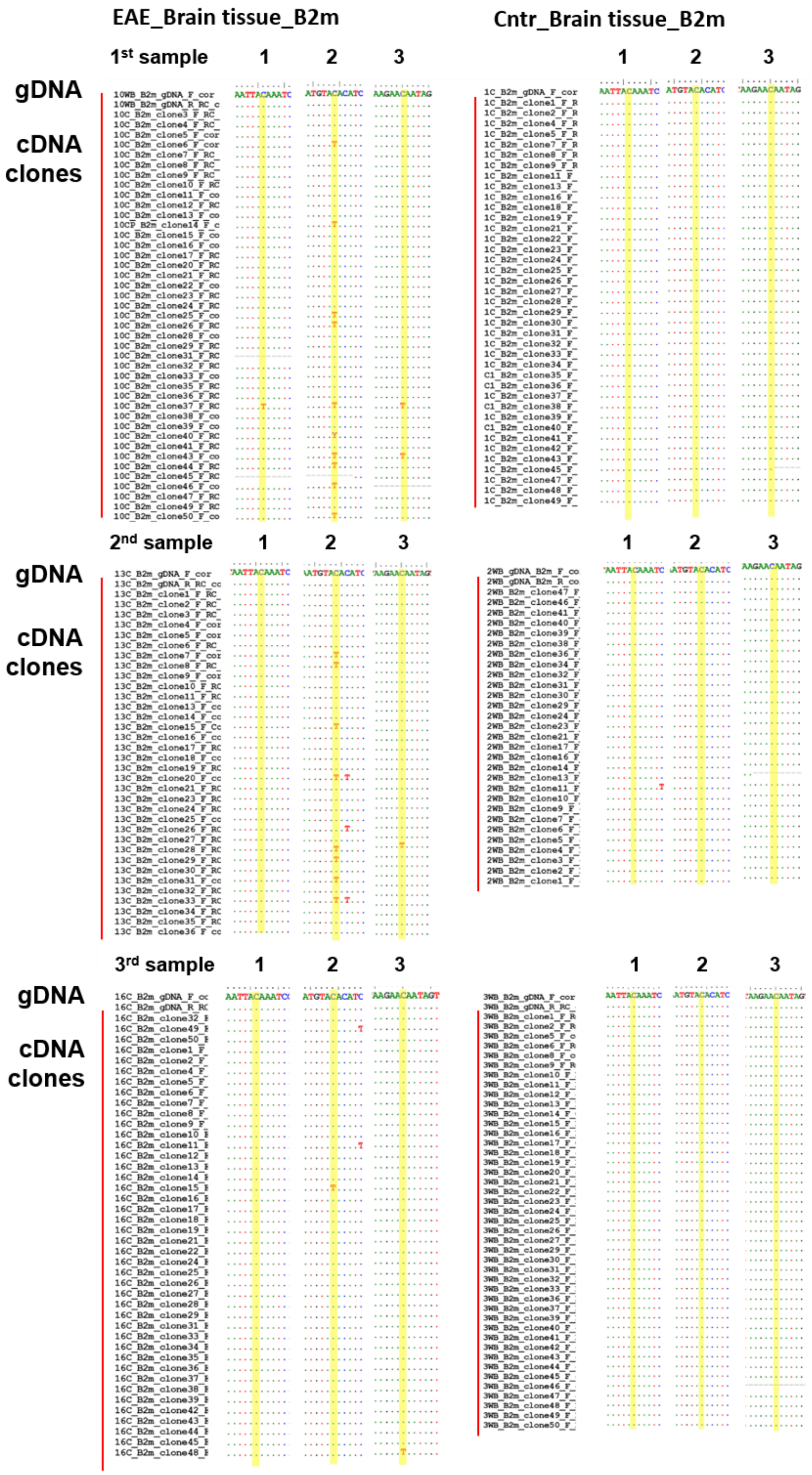

Figure S8. Detailed Sanger sequencing analysis for detection and quantification of C-U-T RNA editing events in the B2m 3'UTR from naïve and acute EAE brain tissue (3 biological replicates). Alignments of cDNA clones relative to the corresponding gDNA. Positions undergoing C-U-T editing as shown in yellow highlight. 1: 2:122152682, 2: 2:122152740, 3: 2:122152871.

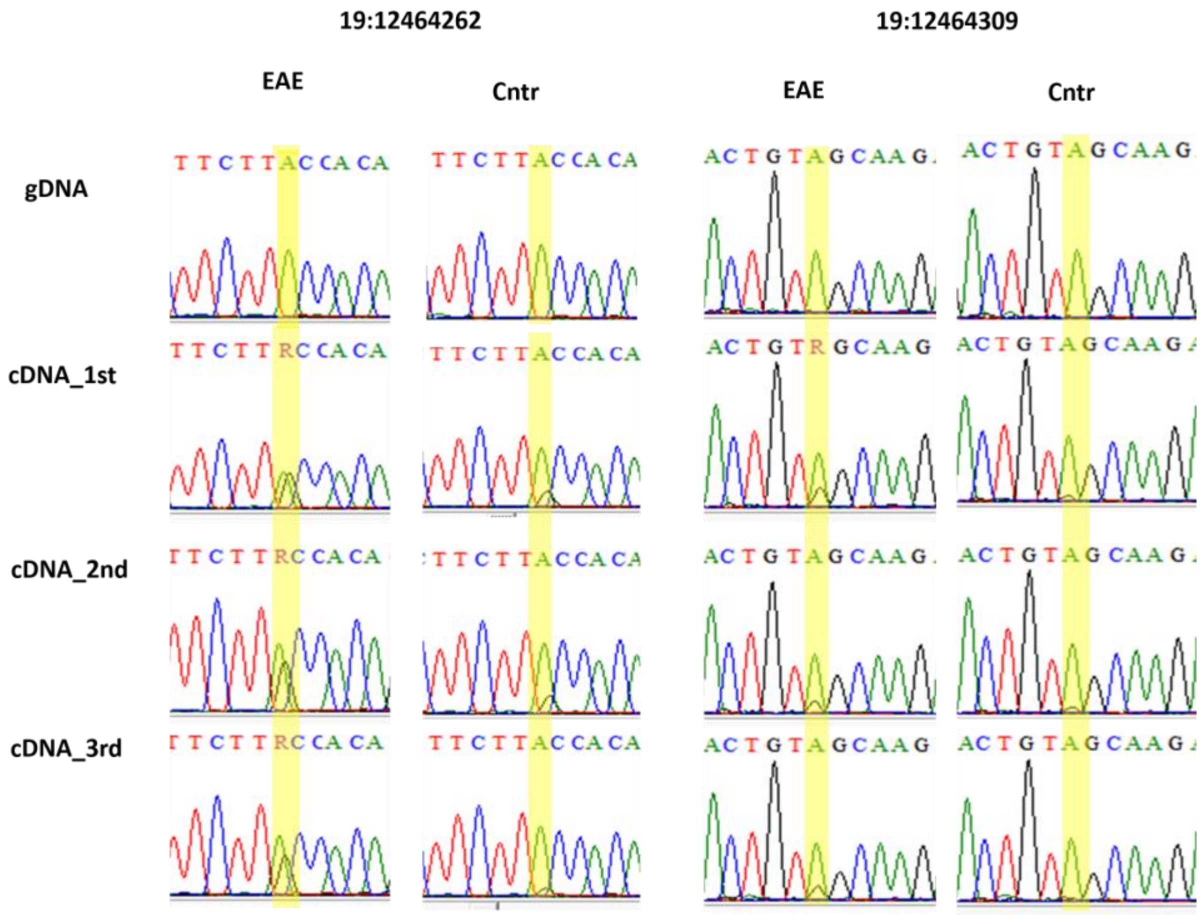

Figure S9: Detailed Sanger sequencing analysis for detection and quantification of A-I-G RNA editing events in the Mpeg1 3'UTR from naïve and acute EAE microglia (3 technical replicates). Chromatogram segments of PCR amplified cDNAs harbouring the editing sites of interest relative and corresponding gDNAs. Positions undergoing A-I-G editing as shown in yellow highlight.

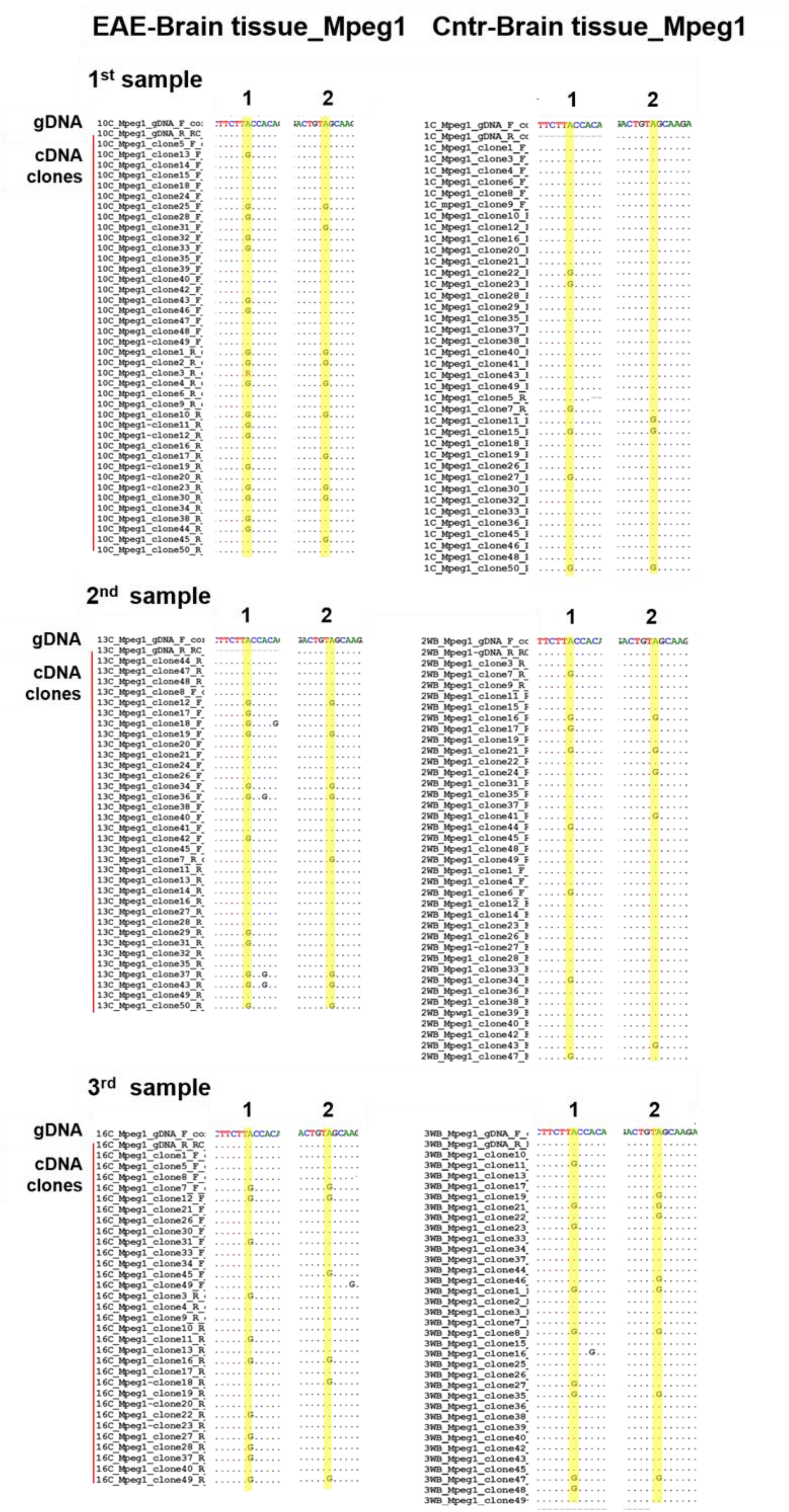

Figure S10. Detailed Sanger sequencing analysis for detection and quantification of A-I-G RNA editing events in the Mpeg1 3’UTR from naïve and acute EAE brain tissue (3 biological replicates). Alignments of cDNA clones relative to the corresponding gDNAs. Positions undergoing A-I-G editing as shown in yellow highlight. 1: 19:12464262, 2: 19:12464309.

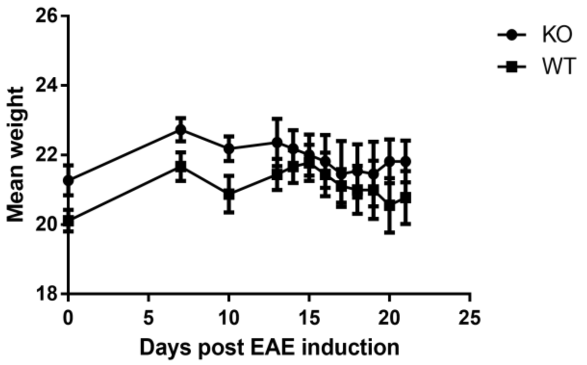

Figure S11. Mean weight variations of Apobec1 KO and WT mice during EAE progression. Significant weight loss differences are observed only close to the end point of the experiment.

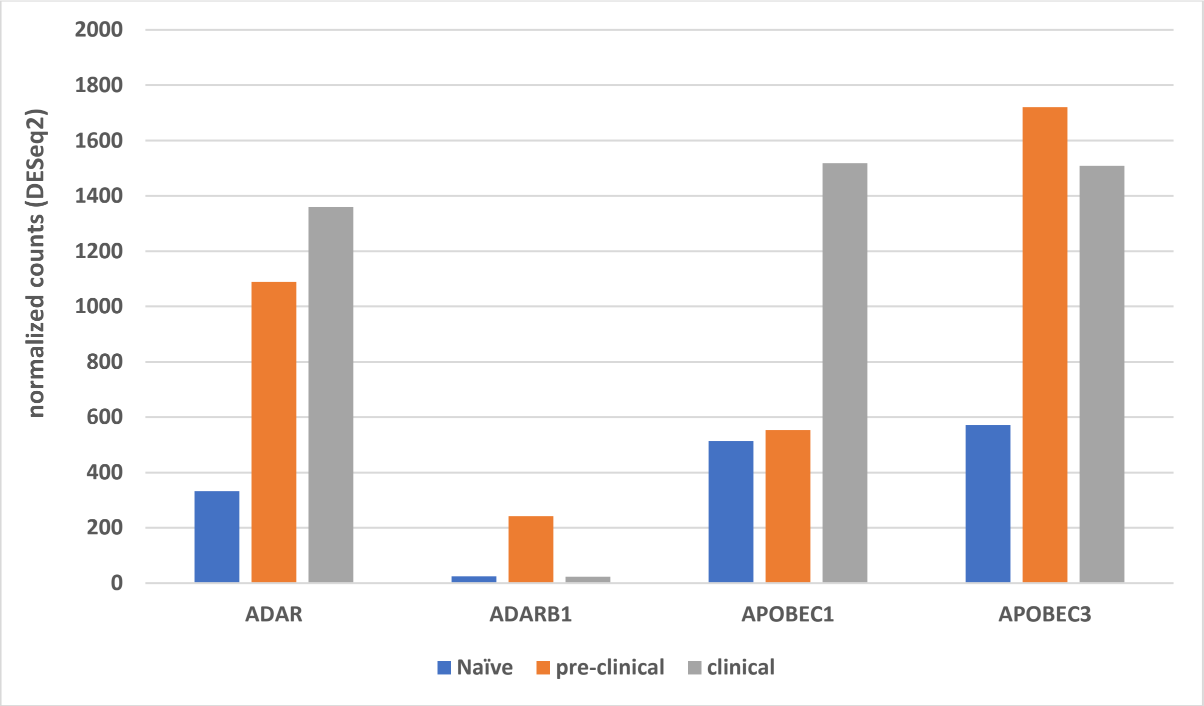

Figure S12. Main RNA-mediating enzymes mRNA expression levels in microglia isolated from naïve, pre-clinical and clinical EAE mice. The bars represent normalized counts (expression levels determined by DESeq2) of ADAR1 (ADAR), ADAR2 (ADARB1), APOBEC1 and APOBEC3 in naïve, pre-clinical and clinical EAE murine microglia (RNA-seq data from Lewis et al., 2014).
